# Rethinking the bodily self: evidence from the enfacement illusion in women at risk for eating disorders

**DOI:** 10.1186/s40337-025-01461-1

**Published:** 2025-12-23

**Authors:** Jade Portingale, Isabel Krug, Tamsyn E. Van Rheenen, Litza Kiropoulos, Cali F. Bartholomeusz, Helen Nasser, David Butler

**Affiliations:** 1 School of Psychological Sciences, The University of Melbourne, Melbourne, VIC 3010 Australia; 2 Department of Psychiatry, Melbourne Medical School, The University of Melbourne, Melbourne, Australia; 3 Centre for Mental Health and Brain Sciences, School of Health Sciences, Swinburne University, Melbourne, Australia; 4 Orygen, Melbourne, Australia; 5 La Trobe University, Melbourne, Australia; 6 Faculty of Psychology and Counselling, The Cairnmillar Institute, Melbourne, Australia

**Keywords:** Eating disorders, Body image, Face perception, Self-perception, Multisensory integration, Enfacement illusion

## Abstract

**Background:**

Bodily illusion research has demonstrated that altered bodily-self-perception in eating disorders (EDs) may be linked to abnormalities in the integration of sensory bodily signals. Experiencing bodily illusions can also temporarily reduce body image disturbance. Whether similar alterations in multisensory integration processes extend to self-face representation and whether face-based multisensory illusions can reduce face or body image disturbance remains unclear. This study investigated whether susceptibility to the enfacement illusion differs based on ED risk status and whether experiencing the illusion reduces face and body image disturbance.

**Methods:**

The sample included 226 women classified as high (*n* = 102) or low (*n* = 124) ED risk, who underwent an enfacement illusion induction procedure involving synchronous (ilillusion-inducing) versus asynchronous (control) visuo-motor stimulation (via facial mimicry) between their own face and an unfamiliar person’s face. Illusion strength was assessed subjectively (via self-report) and objectively (via a self-face recognition task), alongside pre- and post-illusion face and body image outcomes.

**Results:**

Synchronous interpersonal visuo-motor stimulation led to modest changes in self-face recognition (i.e., the other person's face came to be perceived as more similar to one's own); however, these changes were not modulated by ED risk status (high versus low). Cognitive-affective responses to the illusion diverged in unexpected ways. Low ED-risk participants reported reduced body dissatisfaction and dysmorphic concern following synchronous interpersonal visuo-motor stimulation, whilst high ED-risk participants reported increased head and body dissatisfaction following both synchronous and asynchronous stimulation.

**Conclusion:**

These findings suggest that the multisensory processes underlying self-face representation, and ultimately supporting self-recognition and the integrity of self-other boundaries, may not be disrupted in individuals with elevated ED symptomatology. This observation may challenge the notion of a globally disrupted sense of bodily self in EDs, at least with respect to self-face processing. Instead, current results suggest that ED-related body image disturbance may reflect altered higher-order evaluative or affective processing of self-related social information rather than a fundamental deficit in multisensory integration.

**Supplementary Information:**

The online version contains supplementary material available at 10.1186/s40337-025-01461-1.

## Abstract (plain language summary)

People with eating disorders (EDs) often experience a distorted sense of their own body. Researchers have used body illusions—like the "rubber hand illusion", where participants can experience a fake hand as their own—to explore how the brain combines sensory information to create a sense of "this is my body". Some of these illusions can even temporarily improve body image. The current study investigated how a face-based illusion—the “enfacement illusion,” which induces the feeling that another person’s facial features are blended with one’s own—affects how people at high versus low risk for EDs perceive themselves. Both groups experienced the illusion to a similar degree, suggesting that people at higher risk for EDs may not have deficits in basic brain processes that combine sensory signals to recognise their own face. However, cognitive and emotional responses to the illusion differed. After the illusion, low-ED-risk participants felt more satisfied with their physical appearance, whereas high-ED-risk participants felt more dissatisfied. These findings suggest that for people vulnerable to EDs, body image disturbances may not stem from the way that the brain perceives or integrates body-related sensory information. Instead, disturbances may arise from how these individuals interpret and evaluate such information, especially during social interactions involving another person.

**Supplementary Information:**

The online version contains supplementary material available at 10.1186/s40337-025-01461-1.

## Introduction

Body image is broadly defined as the conscious mental representation of one’s own body and is typically parsed into cognitive-affective and perceptual components [1, 2]. The former involves feelings and thoughts towards one’s body, while the latter refers to the accuracy in estimating one’s own body size and shape [1, 2]. Body image disturbance is a defining feature of eating disorders (EDs), implicated in their onset, maintenance, and relapse [3–5]. Historically, research and interventions for EDs have focused on the cognitive-affective components of disturbance, such as dissatisfaction or overvaluation of weight/shape. The perceptual component, often involving visual body size overestimation [6, 7] has received comparatively less attention in empirical research and intervention [4]. This oversight is notable, as perceptual distortions are common and often associated with poorer clinical outcomes for those with EDs [5, 8]. Advancing our understanding of the mechanisms underlying perceptual body image disturbance and how the bodily self is mentally represented more broadly in individuals with EDs remains a critical aim for ED research.

Bodily self-consciousness refers to the ability to perceive, experience, and recognise one’s body as one’s own [9]. Converging neurological, neuroimaging, and psychological evidence highlights how this experience depends on the dynamic interplay between sensory signals originating from outside the body (exteroceptive signals such as vision, touch) and from inside the body, including from the musculoskeletal system (proprioceptive signals related to posture and movement) and visceral organs (interoceptive signals such as cardiac and respiratory rhythms) [10–12]. Through a process termed multisensory integration, whereby the brain combines sensory signals across multiple modalities, a unified representation of the body and its boundaries emerges, providing the foundation for bodily self-consciousness [13–15]. Multisensory integration operates according to the basic principles of temporal and spatial congruency, whereby sensory inputs are more likely to be integrated when they occur at the same time and originate from the same place [16].

Multisensory bodily (hereafter ‘embodiment’) illusions, such as the rubber hand and full-body illusions, provide powerful experimental tools for probing the malleability of bodily self-consciousness [17, 18]. These paradigms reveal how manipulating the integration of current sensory inputs (e.g., visual, tactile, and proprioceptive) can alter bodily self-consciousness, at least temporarily. For example, in the classic rubber hand illusion, synchronised stroking of a visible rubber hand and one’s own hidden hand can induce the illusory experience of ownership over the rubber hand (a phenomenon termed ‘embodiment’) [19]. Based on the same principles, researchers have used full-body illusions to elicit embodiment over an entire artificial body, often using virtual reality, by synchronising its movements or tactile feedback with the participant's own [20, 21]. Asynchronous interpersonal multisensory stimulation (IMS) (i.e., temporally and/or spatially incongruent inputs) typically diminishes or abolishes these effects [19, 22], although some studies suggest that embodiment effects, though typically weaker, still occur under these conditions [23, 24].

Predictive coding theory provides a compelling neurocomputational framework for understanding embodiment illusions. According to this framework, bodily self-perception results from an adaptive process whereby the brain continuously compares incoming sensory evidence with internal models based on expectations (‘priors’) about the causes of sensory input [25, 26]. The system aims to minimise discrepancies between expected and actual sensory data (termed ‘prediction errors’). This can be achieved by updating priors and the internal model (bodily self-representation) to match current sensory input, provided the sensory evidence is deemed reliable (precise). During embodiment illusions, such as the rubber hand illusion, synchronised IMS creates a conflict with the typical experience of seeing one’s own hand receive touch whilst feeling that touch. To minimise the resultant sensory prediction error, the brain adapts by temporarily updating its internal model (bodily self-representation) to include the rubber hand [17, 26].

EDs are associated with disturbances in bodily self-perception, and increasing evidence from embodiment illusions suggests that impairments in multisensory integration play a critical role [27–29]. Within a predictive coding framework, heightened susceptibility to embodiment may reflect aberrant precision-weighting (e.g., visual signals are deemed more reliable than proprioceptive or interoceptive inputs), resulting in erroneous body model updating and distorted bodily self-perception [27–29]. Consistent with this account, individuals with EDs are more susceptible to embodiment illusions, typically the rubber hand illusion, than healthy controls (e.g [22, 23] for a review, see [27]). Most research has focused on anorexia nervosa, though similar patterns have been found in mixed ED samples (i.e., anorexia nervosa, bulimia nervosa, binge-eating disorder, and other specified feeding or eating disorder) [27]. Interestingly, some evidence suggests that embodiment illusion strength is comparable across current and recovered ED patients [30] and correlates positively with ED symptom severity in community samples (e.g [24, 25] suggesting that such disruptions may represent trait-like vulnerabilities for EDs.

Embodiment illusions also hold therapeutic potential for EDs. Embodying an external body or body part that differs in physical appearance from one’s real or perceived body has been shown to update and correct maladaptive body image in individuals with EDs, at least momentarily [27]. For example, experiencing a full-body illusion with a ‘healthy’ weight avatar has been shown to reduce body size overestimation and body dissatisfaction among individuals with anorexia nervosa [31–33]. From a predictive coding perspective, such therapeutic effects may arise because the illusion induces lower-level shifts in perceptual body representations, which come into conflict with higher-level body image representations, prompting the brain to update existing (dysfunctional) beliefs about the body [17, 26, 34].

In some studies, illusion-induced improvements are greatest in those with EDs (compared to controls) or positively correlate with ED symptom severity [31, 35, 36]. This pattern may reflect a greater baseline sensory prediction error (i.e., mismatch between internal body representation and incoming sensory information), thereby increasing the opportunity for illusion-induced recalibration.

To date, ED-related embodiment illusion research has focused almost exclusively on the body (limbs, abdomen, or full-body representations) [27], leaving the face markedly underexamined [37]. This omission is striking, given that the face may be processed through distinct mechanisms due to its unique and central role in self-referential processing (e.g., self-recognition) [17, 38] and social communication, including evaluations of physical attractiveness [39, 40].

Critically, emerging evidence indicates that individuals with EDs and high ED risk, predominantly involving female samples, exhibit face-specific perceptual disturbances, including poorer self-face recognition accuracy [41, 42], overestimation of facial adiposity, and negative cognitive-affective evaluations of their own facial appearance (e.g., dissatisfaction, lower perceived attractiveness) [41]. Positive correlations between self-face perception and broader body dissatisfaction or body image concerns have also been observed [41, 43, 44]. These findings suggest that EDs may not only be associated with face-specific disturbances in body image, but with wider disruptions in self-representation.

The enfacement illusion extends embodiment principles to self-face representation. Through synchronous visuo-tactile or visuo-motor IMS—such as watching another person's face being stroked or performing facial movements in synchrony with one's own—participants can experience perceptual shifts in self-other boundaries, so that the other's face is assimilated into their own self-representation [45–47]. This manifests as increased perceived self-resemblance to the 'other' and behavioural shifts in self-face recognition thresholds [45–47]. Heightened susceptibility to the enfacement illusion in ED populations may reflect disruptions in self-referential processing that are linked to underlying abnormalities in multisensory integration. Analogous to findings from embodiment illusions, experiencing the enfacement illusion may also reduce body image disturbance, particularly in face-specific domains.

### The current study

Considering both the growing research applying embodiment illusions to the study and treatment of bodily self-perception disturbances in EDs [27] and accumulating evidence of self-face perceptual disturbances in EDs [41], critical gaps remain: whether face-specific multisensory integration mechanisms are altered in ED-vulnerable individuals and whether updating self-face representation through a multisensory-based intervention can improve face and body image. Building on recommendations by [37], this study is the first to integrate these parallel lines of research by applying the enfacement illusion to individuals with ED symptomatology. The study addressed three core research questions:

1. Does susceptibility to the enfacement illusion differ between high- and low-ED-risk individuals?
2. Does experiencing the enfacement illusion reduce face and body image disturbance across perceptual (adiposity estimation) and cognitive-affective (dissatisfaction, attractiveness, concern) domains?
3. Does ED risk status (high versus low) moderate such effects?

We hypothesised that individuals at high (relative to low) ED risk would show greater susceptibility to the enfacement illusion (H1), experiencing the enfacement illusion would reduce face and body image disturbance (H2), and that reductions would be more pronounced in individuals at high (relative to low) ED risk (H3).

To address the current aims, we recruited a community-based sample of cisgender females stratified into high- and low-ED-risk groups based on a validated self-report screening tool. We focused on females, given the higher prevalence of EDs in this group [48] and the predominance of face-related research in female populations [41–43].

Facial models used to induce the illusion were pre-rated as average in perceived facial attractiveness, likeability, and adiposity. These attributes were selected based on evidence that the model’s attractiveness and likeability influence enfacement strength [49, 50] and evidence that adiposity is robustly linked to perceived facial attractiveness [39]. Moreover, individuals with elevated ED symptoms tend to demonstrate negatively biased self-face perception [41]; hence, average faces may be perceived as favourable and attainable, and thus, elicit positive therapeutic effects. This strategy aligns with previous full-body illusion research demonstrating that embodying average or same-sized avatars can reduce body image disturbance in ED populations [27].

## Method

### Participants

This study was pre-registered [51]. Ethical approval was granted by the University of Melbourne's local ethics committee (ID: 2056250.1). The study adhered to the protocol outlined in Portingale et al. [52]. Participants were required to be English-speaking, cisgender females, at least 18 years old, and self-identify as either White or East/Southeast Asian. Ethnic groups were matched to the stimulation models (see Stimuli section). Participants were not excluded based on psychiatric comorbidities (e.g., anxiety, depression, or autism-related traits) to capture the natural clinical heterogeneity of ED presentations and enhance ecological validity [53].

Participants were recruited via multiple sources across Australia, including the undergraduate psychology research participation pool at the University of Melbourne, noticeboards at the University of Melbourne and Cairnmillar Institute, social media, ED-related settings (e.g., organisation websites, social media pages, private practice), and personal networks. Recruitment was conducted in two phases. Initially, the sample was drawn primarily from the university research program. However, this group under-represented individuals with elevated ED symptoms relative to community estimates. Consequently, a pre-screening protocol using the Eating Attitudes Test-26 (EAT-26 [46]) was introduced mid-study to increase the representation of high-ED-risk individuals. In the second recruitment phase, only participants scoring ≥ 20 on the EAT-26 were invited to participate in the experimental procedure. All participants, regardless of recruitment phase, underwent identical testing.

A total of 226 female participants were recruited and stratified into high (*n* = 102, 45.1%) or low (*n* = 124, 54.9%) ED risk groups based on the EAT-26 [54] (≥ 20 for high risk, < 20 for low risk). A score of 20 or higher indicates clinically significant levels of ED symptoms, regardless of lifetime ED history. Participants were predominantly young adults (*M* age = 22.8 years, *SD* = 6.2) within the healthy BMI range. In the low ED-risk group, 15 participants (12.1%) were underweight and 19 (15.3%) were overweight or obese. In the high ED-risk group, 17 participants (16.7%) were underweight and 19 (18.6%) were overweight or obese.

Compensation varied by phase completion: university research program participants received 0.5–2 course credits, while community participants received AUD$30–40 in e-gift cards or were entered into a draw to win one of five $50 e-gift cards.

### Stimuli

#### Face models

Ten unfamiliar young adult female models (six White, four East/Southeast Asian; age ≈ 20–25 years) were used to create the experimental stimuli displaying the unfamiliar face (Other). To obtain these models, in a formal pilot validation study, an independent community sample of 60 young adult women (30 White, 30 East/Southeast Asian) rated ethnicity-matched neutral-expression face images (10 White, 10 East/Southeast Asian) on physical attractiveness, adiposity, and likeability using 7-point Likert scales (e.g., -3 = *very unattractive*, 3 = *very attractive*). Final models were selected for average ratings (e.g., 0 = *neither unattractive nor attractive*) across the three attributes: attractiveness (*M* ≈ 0.07, *SD* ≈ 1.24), adiposity (*M* ≈ -0.13, *SD* ≈ 0.63), and likeability (*M* ≈ 0.21, *SD* ≈ 1.25) (see Supplementary Table S1 for full results of the pilot study). This approach was informed by evidence that interpersonal perceptions, including physical attractiveness [45, 49] and likeability [50], influence enfacement strength, and that facial adiposity is linked to perceived attractiveness [43]. Neutral emotional expressions were verified by pilot raters. Note, whilst six models (3 White, 3 East/Southeast Asian) were validated in the current pilot study, the remaining four models (3 White, 1 East/Southeast Asian) were adopted from previous research by our group; however, complete pilot data for these models are unavailable. All models were recruited from the same community (Melbourne, Australia), matched on age range, and all model stimuli (outlined below) were created under identical conditions.

#### Interpersonal multisensory stimulation videos

**Fig. 1:**
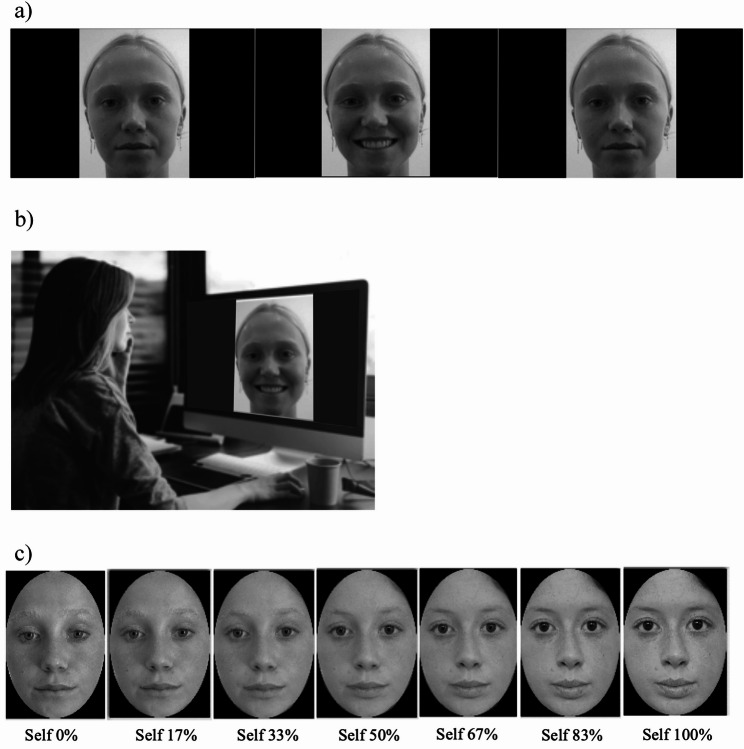
Experimental Stimuli and Set-Up. **a** An interpersonal visuo-motor stimulation video depicting a model alternating between neutral and smiling expressions at 10-second intervals across the 150-second trial; **b** participant completing the asynchronous stimulation (facial mimicry) condition, performing the opposite facial expression to the model (neutral versus smiling expression); and (**c**) self-face recognition task stimuli showing morphed images from 100% Other (0% Self) to 0% Other (100% Self) in standardised 17% increments for illustrative purposes

Prior to the study, videos were recorded of each model to develop the facial mimicry task used for visuo-motor IMS (i.e., enfacement illusion induction) (Fig. 1a–b). Each video displayed a model’s face (Other)—matching the participant’s sex and ethnic group—alternating between a neutral expression and an exaggerated visible-tooth smile every 10 s while maintaining a direct gaze with the camera. Each video lasted 150 s, slightly longer than the typical 120 s in prior studies [45, 47, 55], to increase the likelihood of eliciting enfacement. Videos were converted to greyscale against a black background to minimise visual distraction. Faces occupied approximately 40% of the horizontal video dimension. All videos were created by the lead researcher (J. P.) and closely monitored in real time to ensure uniformity (e.g., in emotional expression quality). All stimuli were approved by the research team for clarity and consistency (see Supplementary Figure S1 for example model images).

#### Face morphing videos

A neutral-expression photograph was taken of each final model’s face prior to the study and of each participant’s face at the beginning of each experimental session. All images were standardised (prior to the study during stimulus preparation for the models and during the study for the participants): non-facial features (i.e., ears, hair, and background) were removed using an oval template, a black template was added, and images were converted to greyscale using PhotoScape X (Version 4). The participant’s images were mirror-transposed.

Next, a computerised morphing procedure implemented using FantaMorph software [56], employed a mesh warping algorithm to merge each participant’s face with the face of their assigned sex- and ethnicity-matched Other (the same face used in the IMS video). The video sequence contained 100 frames that transitioned from 100% Other (0% Self) to 0% Other (100% Self) in the Other to Self direction. The morphed videos were 100 s in duration and progressed at a rate of 1% change in face per second, resulting in a gradual blending of the two faces. These videos were used to generate the self-face recognition task (Fig. 1c), which measured objective enfacement (see Measures).

### Measures

#### Demographic and trait-based

*Eating Disorder Symptomatology* The 26-item Eating Attitudes Test (EAT-26) [54] is among the most widely used measures of ED symptomatology and serves as a screening tool for clinical and community populations (e.g [57, 58]). All responses on a 6-point scale are summed (range: 0–78), with higher scores indicating greater pathology and cut-off scores of ≥ 20 are used to identify individuals at high risk of EDs. Internal consistency in the current study was excellent (Cronbach’s α = 0.93).

*Alexithymia Symptomatology* We controlled for alexithymia, a common emotion processing deficit observed in ED populations [59] that has been shown to influence embodiment illusion susceptibility [60]. The Toronto Alexithymia Scale (TAS-20) [61] assesses alexithymia using 20 items rated on a 5-point scale. All responses are summed (range: 20–100), with higher scores indicating greater alexithymia symptoms. Internal consistency was modest in the current study (Cronbach’s α = 0.65).

*Demographics* Self-report information included age, height, weight (to calculate BMI; kg/m^2^), ethnicity, primary language spoken at home, sexual orientation, marital status, lifetime ED diagnosis and subtypes.

#### State-based experimental

*Subjective Enfacement* The enfacement questionnaire [62] assesses subjective enfacement across 8 items rated on a 7-point scale (-3 = *strongly disagree*, 3 = *strongly agree*). The scale includes three subcomponents: *self-identification* (4 items; feeling that the model’s face was their own), *similarity* (2 items; perceived resemblance), and *affect* (2 items; perceived attractiveness/trustworthiness of the model). Subcomponent scores are calculated by averaging the relevant items (range: -3 to 3), and the total score is the average of the three subcomponent scores (range: -3 to 3). Higher scores indicate greater subjective experience of the illusion. In the present study, internal consistency was good across time-points (Cronbach’s α range: 0.82–0.85).

*Objective Enfacement* Using a self-face recognition task [46], participants viewed a video morphing from 100% Other-face (0% Self) to 100% Self-face (0% Other) and were instructed to stop playback by pressing the space bar with their left index finger at the exact moment the image appeared more like “Self” than “Other”. Stopping times (range: 0–100 s) were converted to a percentage of Self (e.g., 60 s = 60% Self). Earlier stopping times (lower % Self) indicate increased assimilation of the Other into one's self-face representation. 

#### State-based face and body image disturbance

*Facial Attractiveness and Adiposity* Single-item 7-point scales assess current self-perceived facial attractiveness (0 = *very unattractive*; 3 = *neither unattractive nor attractive;* 6 = *very attractive*) and facial weight (0 = *very underweight*; 3 = *neither under or overweight*; 6 = *very overweight*). These single-item measures are consistent with widely used methodologies in prior research assessing self-perceived facial attractiveness and adiposity [39, 63, 64]. The psychometric properties of the current facial attractiveness scale have been supported [65], and Likert-type ratings of facial adiposity are robustly related to objective indices (i.e., BMI), supporting their construct validity [39]. Higher scores indicate greater perceived facial attractiveness and adiposity.

*Head and Body Dissatisfaction* The Body Satisfaction Scale (BSS) [66] is a 16-item measure assessing satisfaction with specific body parts. The scale comprises two validated 7-item subscales: ‘head’ satisfaction (head, face, jaw, teeth, nose, mouth, eyes) and ‘body’ satisfaction (shoulders, chest, tummy, arms, hands, legs, feet). Although originally validated as a trait measure, we adapted the phrasing to assess momentary satisfaction (i.e., “How satisfied are you *right now*...”) on a 7-point scale (1 = *very satisfied*; 7 = *very dissatisfied*), consistent with previous experimental body image research (e.g., α = 0.91 [67]). Scores range from 7 to 49 per subscale, with higher scores indicating greater dissatisfaction. In the current study, internal consistency was good to excellent for both sub-scales across time-points (Cronbach’s α range: 0.85– 0.91).

*Dysmorphic Concern* The Body Image Concern Inventory (BICI-10) [68] is a 10-item measure assessing dysmorphic appearance concern on a 5-point scale (1 = *strongly disagree*; 5 = *strongly agree*). Although originally validated as a trait measure, we adapted the phrasing to assess momentary concern (i.e., “Please indicate how each feeling or behaviour relates to you *in this exact moment*”). Scores range from 10 to 50, with higher scores indicating greater concern. In the current study, internal consistency was excellent across time-points (Cronbach’s α range: 0.91–0.94).

### Design and experimental procedure

**Fig. 2:**
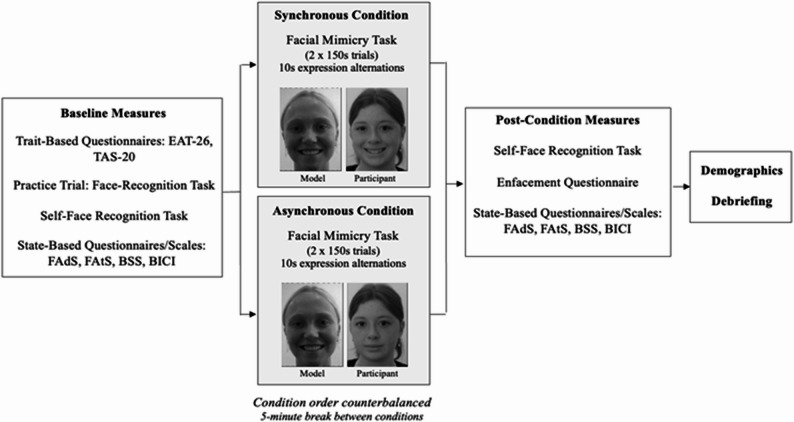
Graphical Representation of the Experimental Procedure. *EAT-26* Eating Attitudes Test-26 item, *TAS-20* Toronto Alexithymia Scale-20 item, *FAds* Facial Adiposity Scale, *FAts* Facial Attractiveness Scale, *BSS* Body Satisfaction Scale, *BICI* Body Image Concern Inventory

Figure 2 provides an overview of the experimental procedure. The study employed a within-subjects design with a baseline condition and two experimental conditions comprising (i) synchronously- and (ii) asynchronously-timed visuo-motor IMS involving facial mimicry. The choice to induce the enfacement illusion using facial mimicry rather than classic visuo-tactile stroking methods (e.g [46]) was necessitated by COVID-19 pandemic-related restrictions precluding face-to-face procedures. Nonetheless, emerging evidence supports enfacement induction through visuo-motor coherence involving the mimicry of facial or head movements [55, 69, 70].

Each participant completed a one-on-one experimental session with a trained researcher over Zoom videoconferencing via their laptop/computer, lasting approximately 1.5–2 h. At the beginning of the session, participants confirmed all requirements for set-up (i.e., seated comfortably at eye-level in front of their laptop/computer screen, at a viewing distance of approximately 50 cm, in a quiet, well-lit room). The baseline phase began with measures of trait-based psychopathology (EAT-26, TAS-20), followed by a practice face recognition task using morphed celebrity facial stimuli (George Clooney and Brad Pitt) to ensure a comprehensive understanding of the upcoming self-face recognition task. Participants then completed the baseline self-face recognition task, which involved viewing the morphing video that changed from 100% Other-face to 100% Self-face. Participants were instructed to keep their gaze and attention fixated on the screen. The stopping time was recorded by the researcher and participants were not permitted to alter their decision. The self-face recognition task was followed by state-based face and body image measures (facial attractiveness/adiposity, BSS, BICI-10), presented in a random order. The baseline assessment lasted approximately 20 min.

Participants then completed two IMS conditions: one with synchronous visuo-motor IMS (experimental condition) and one with asynchronous visuo-motor IMS (control condition); the order of conditions was counterbalanced across participants. In the synchronous condition, participants viewed the pre-recorded IMS video of a model alternating between neutral and smiling facial expressions (every 10 s) per 150-second trial and were instructed to mimic the model’s expressions in real time (e.g., smile when the model smiled). Each participant was randomly assigned to one ethnicity-matched model—given ethnicity’s influence on self-other processes, including mimicry [71] and embodiment [72]—which was kept consistent across the mimicry and self-face recognition tasks. In the asynchronous condition, participants viewed the same video but were instructed to perform the opposite expression (e.g., smile when the model showed a neutral expression). Each condition involved two 150-second mimicry trials. Instructions were read aloud by the experimenter, and participants were required to verbally confirm their understanding before commencing. The experimenter continuously monitored participant engagement via Zoom (e.g., expression accuracy and gaze direction).

Following completion of both trials in each IMS condition, participants completed measures of objective enfacement (the same self-face recognition task as at baseline), subjective enfacement (enfacement questionnaire), and state-based body and face image (facial attractiveness/adiposity, BSS, BICI-10; presented in a random order). Each condition lasted approximately 15–20 min. A mandatory 5-minute break between conditions was included to minimise carryover effects while maintaining environmental consistency.

Upon completing both conditions, participants provided demographic information (approximately 10 min) and were then debriefed. All questionnaires and rating scales were administered via Qualtrics, with survey links shared via the Zoom chat. The self-face recognition and mimicry tasks were delivered via PowerPoint: participants opened the file locally, presented it in full-screen, and shared their screen for monitoring.

### Planned statistical analyses

Multilevel modelling (MLM) replaced the pre-registered repeated measures ANCOVA to better accommodate the nested data structure (repeated measurements within participants). All analyses used restricted maximum likelihood estimation (REML) with random intercepts at the participant level, conducted in R Studio (v.4.4.1) using lme4 and lmerTest packages [73, 74]. All models included age, BMI, ethnicity, and alexithymia as covariates, given their established relationship with ED symptomatology and bodily self-perception [59, 75, 76].

To test Hypothesis 1 (ED risk differences in enfacement susceptibility), subjective enfacement was assessed using the enfacement questionnaire (total and subscale scores). MLMs included fixed effects for Time (synchronous vs. asynchronous), Group (low vs. high ED risk), and their interaction. Higher scores in the synchronous versus asynchronous condition indicated subjective enfacement. Objective enfacement was assessed using the self-face recognition task (% Self-scores at stopping time). MLMs included fixed effects for two Time contrasts (baseline vs. synchronous; baseline vs. asynchronous), Group, and their interactions. Following established enfacement methodology [77–80], these baseline-referenced contrasts provide the most appropriate assessment of perceptual shifts. Earlier stopping times (lower % Self-scores) post-IMS relative to baseline indicate increased acceptance of the other’s facial features as self, with the asynchronous condition serving as a control [77–80]. The synchronous versus asynchronous contrast was explored in the Supplementary Materials.

To test Hypotheses 2–3 (face/body image disturbance changes and ED risk moderation), separate MLMs were conducted for perceived facial attractiveness, perceived facial adiposity, head dissatisfaction, body dissatisfaction, and dysmorphic concern. Fixed effects included two Time contrasts (baseline vs. synchronous; baseline vs. asynchronous), Group, and their interactions. Reductions in face/ body image disturbance were indicated by lower scores post-IMS versus baseline for all measures except attractiveness (where higher scores reflected reduced disturbance).

Significant interactions were probed using Bonferroni-adjusted pairwise comparisons and estimated marginal means. Semi-partial *R*² values were computed using the Nakagawa and Schielzeth method. Model fit was assessed via conditional *R*² (variance explained by fixed and random effects) and marginal *R*² (fixed effects only), interpreted cautiously, given methodological variability [81].

An a priori power analysis for the originally planned ANCOVA indicated *N* = 159 was required to detect medium-to-large effects with >80% power. A priori power estimation for MLM is less standardised, as it depends on several parameters that are difficult to estimate in advance [82]. Post hoc power analysis using ‘pwr’ in R Studio confirmed that the final sample showed high power (>0.99) for detecting medium (f² ≈ 0.13) and large (f² ≥ 0.25) fixed effects across all dependent variables, consistent with effect sizes reported in prior embodiment research in ED populations [27]. Conditional *R*² estimates showed high power (1.00) even for smaller effects (f² = 2.10–17.87).

Exploratory analyses examined whether aspects of the model used, including (i) model identity (coded uniquely for each of the 10 models), (ii) timing of model validation (binary-coded: current versus previous), and (iii) participants’ perceived model attractiveness, influenced outcomes. These variables were assessed as additional covariates and the findings are reported in the Results (see Supplementary Material for details).

## Results

### Assumptions checking

MLM assumptions were rigorously checked. Shapiro-Wilk tests indicated normality violations across all models, though visual inspections showed relatively normal distributions. Transformations (log and square) and outlier removal did not resolve these issues. To ensure robustness, we conducted bootstrapping for all models (1000 samples, bias-corrected and accelerated 95% confidence intervals) and REML estimation, which enhances reliability under these conditions. Homoscedasticity was confirmed, and multicollinearity (Variance Inflation Factor scores) was satisfactory for all models. Intraclass correlation coefficients (0.68–0.93) supported MLM. All bootstrapped results confirmed the patterns observed in the original analyses (see Supplementary Material for a summary of the findings).

### Demographic and clinical characteristics

Detailed participant demographic and clinical characteristics are displayed in Table 1. Compared to the low ED-risk group, participants in the high ED-risk group were significantly older, had higher education levels, and reported more lifetime ED diagnoses, as well as higher levels of ED and alexithymia symptomatology. High- and low-ED-risk groups did not significantly differ in terms of BMI, ethnicity, primary language, sexual orientation, or marital status.

**Table 1:** Demographic and clinical characteristics for full sample and by eating disorder risk group

Demographic Variable	Comparison: Eating Disorder Risk				
	Low Risk ^a^ (*n* = 124, 54.87%)	High Risk ^b^ (*n* = 102, 45.13%)	Total (*N* = 226)	*t*/χ^2^	*p*
Age (*M* ± *SD*)	21.7 ± 4.69	24.0 ± 7.40	22.76 ± 6.16	-2.78	**0.006**
BMI (*M* ± *SD*)	21.8 ± 3.22	22.2 ± 4.54	21.97 ± 3.87	-0.84	0.400
BMI categories (*n*, %) ^c^				3.01	0.389
Underweight	15 (12.1%)	17 (16.7%)	32 (14.2%)		
Healthy weight	90 (72.6%)	66 (64.7%)	156 (69.0%)		
Overweight	16 (12.9%)	13 (12.7%)	29 (12.8%)		
Obese	3 (2.4%)	6 (5.9%)	9 (4.0%)		
Ethnicity (*n*, %)				0.05	0.820
White	48 (38.7%)	41 (40.2%)	89 (39.4%)		
Asian (East/Southeast)	76 (61.3%)	61 (59.8%)	137 (60.6%)		
Primary language (*n*, %)				0.01	0.932
English	70 (56.5%)	57 (55.9%)	127 (56.2%)		
Other	54 (43.5%)	45 (44.1%)	99 (43.8%)		
Highest education completed (*n*, %)				15.44	**0.001**
Year 12 or below	2 (1.6%)	2 (2.0%)	4 (1.8%)		
Certificate/diploma	81 (65.3%)	54 (52.9%)	135 (59.7%)		
Bachelor’s degree	25 (20.2%)	36 (35.3%)	61 (27.0%)		
Postgraduate degree (e.g., Honours, Masters, PhD)	15 (12.1%)	10 (9.8%)	25 (11.1%)		
Sexual orientation (*n*, %)				1.42	0.922
Heterosexual	89 (71.8%)	71 (69.6%)	160 (70.8%)		
Homosexual	4 (3.2%)	4 (3.9%)	8 (3.5%)		
Bisexual	22 (17.7%)	21 (20.6%)	43 (19.0%)		
Asexual	3 (2.4%)	3 (2.9%)	6 (2.7%)		
Other	5 (4.0%)	3 (2.9%)	8 (3.5%)		
Prefer not to say	1 (0.8%)	0 (0.0%)	1 (0.4%)		
Marital status (*n*, %)				3.15	0.534
Single	77 (62.1%)	65 (63.7%)	142 (62.8%)		
In a relationship	35 (28.2%)	31 (30.4%)	66 (29.2%)		
Married	6 (4.8%)	2 (2.0%)	8 (3.5%)		
De facto	6 (4.8%)	3 (2.9%)	9 (4.0%)		
Separated	0 (0.0%)	1 (1.0%)	1 (0.4%)		
Lifetime eating disorder diagnosis (*yes*, %)	6 (4.8%)	34 (33.3%)	40 (17.7%)	31.20	**< 0.001**
Eating disorder diagnosis status (*yes*, %)				6.77	**0.034**
Current	0 (0.0%)	19 (18.63%)	19 (8.41%)		
Recovered	5 (4.03%)	11 (10.78%)	16 (7.08%)		
Lifetime	1 (0.81%)	4 (3.92%)	5 (2.21%)		
Lifetime eating disorder diagnosis type (*yes*, %)				2.94	0.817
Anorexia nervosa (restricting)	4 (3.23%)	13 (12.75%)	17 (7.52%)		
Anorexia nervosa (binge-purge)	1 (0.81%)	5 (4.90%)	6 (2.65%)		
Bulimia nervosa (purging)	0 (0.0%)	3 (2.94%)	3 (1.33%)		
Binge eating disorder	1 (0.81%)	5 (4.90%)	6 (2.65%)		
EDNOS/OSFED	0 (0.0%)	5 (4.90%)	5 (2.21%)		
Other	0 (0.0%)	3 (2.94%)	3 (1.33%)		
Eating disorder symptomatology ^d^ (*M* ± *SD*)	6.75 ± 5.15	32.74 ± 12.09	18.48 ± 15.75	-20.24	**< 0.001**
Alexithymia ^e^ (*M* ± *SD*)	48.0 ± 11.4	55.6 ± 10.7	51.45 ± 11.69	-5.17	**< 0.001**

*BMI* Body Mass Index (kg/m^2^), *EDNOS* eating disorder not otherwise specified, *OSFED* other specified feeding and eating disorder, *M* mean, *SD* standard deviation. *t*-test for continuous variables, chi-squared test for categorical variables

^a^EAT-26 (Garner et al., 1982) scores below 20

^b^EAT-26 scores at or above 20

^c^BMI categories: 0 = 0–18.49, 1 = 18.50–24.99, 2 = 25–29.99, 3 = 30–60

^d^EAT-26 score (continuous)

^e^Total TAS-20 (Bagby et al., 1994) scores. Significant (two-sided) *p* values (< 0.05) are bolded

### Susceptibility to the enfacement illusion

Descriptive statistics for all outcome measures are displayed in Table 2, MLM analyses in Table 3, and post-hoc analyses in Table 4.

**Table 2:** Descriptive statistics for total sample and by eating disorder risk group

Outcome Variable	Timing Condition	Total Sample (*N* = 226)			Low ED Risk (*n* = 124)			High ED Risk (*n* = 102)			
		*M*	*SD*	Observed range	*M*	*SD*	Observed range	*M*	*SD*	Observed range	Possible range
Subjective enfacement											
Self-identification	Sync	-1.27	1.47	-3.00–3.00	-1.41	1.42	-3.00–2.00	-1.11	1.50	-3.00–3.00	-3.00–3.00
	Async	-1.77	1.31	-3.00–2.50	-1.93	1.18	-3.00–1.50	-1.57	1.44	-3.00–2.50	
Similarity	Sync	-0.73	1.83	-3.00–3.00	-0.84	1.85	-3.00–3.00	-0.60	1.82	-3.00–3.00	-3.00–3.00
	Async	-0.90	1.79	-3.00–3.00	-0.95	1.75	-3.00–3.00	-0.83	1.83	-3.00–3.00	
Affect	Sync	-0.11	1.85	-3.00–3.00	0.00	1.81	-3.00–3.00	-0.23	1.91	-3.00–3.00	-3.00–3.00
	Async	-0.33	1.83	-3.00–3.00	-0.14	1.67	-3.00–3.00	-0.57	1.99	-3.00–3.00	
Total	Sync	-0.70	1.35	-3.00–2.67	-0.75	1.36	-2.92–2.17	-0.64	1.34	-3.00–2.67	-3.00–3.00
	Async	-1.01	1.25	-3.00–2.08	-1.10	1.16	-3.00–1.83	-0.99	1.35	-3.00–2.17	
Objective enfacement	Baseline	61.86	15.31	10.54–96.09	61.77	14.69	29.09–94.95	61.96	16.10	10.54–96.09	0–100
	Sync	56.83	15.41	14.50–94.81	56.06	15.29	26.33–93.36	57.77	15.58	14.50–94.81	
	Async	57.23	15.88	21.82–100	57.13	15.82	21.82–99.41	57.35	16.03	22.20–100	
Facial attractiveness	Baseline	2.60	1.42	0–6	2.93	1.35	0–6	2.21	1.42	0–6	0–6
	Sync	2.63	1.45	0–6	2.99	1.38	0–6	2.20	1.41	0–6	
	Async	2.52	1.47	0–6	2.86	1.38	0–6	2.12	1.49	0–6	
Facial adiposity	Baseline	3.74	0.94	1–6	3.55	0.86	1–6	3.98	0.98	2–6	1–6
	Sync	3.71	0.96	1–6	3.52	0.81	2–6	3.94	1.08	1–6	
	Async	3.73	0.93	1–6	3.53	0.80	1–6	3.98	1.02	1–6	
Head dissatisfaction	Baseline	24.59	9.34	7–49	21.97	8.96	7–49	27.78	8.83	9–46	7–49
	Sync	24.88	10.77	7–49	21.28	10.09	7–49	29.25	9.97	7–49	
	Async	25.08	10.77	7–49	21.55	9.58	7–49	29.38	10.61	7–49	
Body dissatisfaction	Baseline	26.61	9.89	7–49	23.14	9.29	7–49	30.83	8.94	9–48	7–49
	Sync	26.61	10.97	7–49	22.33	10.06	7–49	31.81	9.75	10–49	
	Async	26.57	10.88	7–49	22.46	9.73	7–49	31.57	10.11	9–49	
Dysmorphic concern	Baseline	35.73	10.15	11–50	31.27	9.66	11–50	41.15	7.85	16–50	10–50
	Sync	34.88	11.71	10–50	29.73	11.25	10–50	41.15	8.90	10–50	
	Async	35.12	11.15	10–50	30.36	10.434	10–50	40.91	9.08	11–50	

Objective enfacement scores show the mean % of frames in the morphed video for which the face was perceived as looking more like ‘self’ than ‘other’

*ED* eating disorder; *M* mean; *SD* standard deviation; *sync* synchronous; *async* asynchronous

**Table 3:** Fixed effects results for all outcome Variables, random effects, and model fit

Outcome Variable	Fixed Effects	b	t	*p*	b Bootstrapped 95% CI (LB, UB)	Semi-partial *R*²	Random Effects (Variance)		Conditional *R*²	Marginal *R*²	ICC
							Intercept	Residual			
Subjective enfacement											
Self-identification	Time: async	-0.51	-4.79	**< 0.001**	[-0.72, -0.30]	0.019	1.14	0.72	0.65	0.09	0.61
	ED risk: high	0.33	1.70	0.090	[-0.04, 0.72]	0.007					
	Age	-0.04	-2.62	**0.009**	[-0.06, -0.01]	0.024					
	BMI	-0.01	-0.49	0.622	[-0.06, 0.03]	0.001					
	Ethnicity: Asian	-0.38	-2.21	**0.028**	[-0.73, -0.05]	0.017					
	Alexithymia	0.01	1.22	0.226	[-0.01, 0.02]	0.005					
	Time: async × ED risk: high	0.04	0.27	0.786	[-0.25, 0.36]	0.000					
Similarity	Time: async	-0.11	-0.89	0.376	[-0.34, 0.12]	0.001	2.14	0.95	0.72	0.08	0.69
	ED risk: high	0.29	1.15	0.251	[-0.18, 0.76]	0.003					
	Age	-0.05	-2.82	**0.005**	[-0.09, -0.02]	0.029					
	BMI	-0.03	-0.92	0.357	[-0.08, 0.03]	0.003					
	Ethnicity: Asian	-0.75	-3.33	**0.001**	[-1.21, -0.30]	0.040					
	Alexithymia	0.01	1.14	0.257	[-0.01, 0.03]	0.005					
	Time: async × ED risk: high	-0.12	-0.65	0.518	[-0.47, 0.23]	0.000					
Affect	Time: async	-0.13	-1.11	0.267	[-0.37, 0.09]	0.001	2.25	0.89	0.74	0.09	0.72
	ED risk: high	-0.24	-0.97	0.332	[-0.74, 0.23]	0.002					
	Age	0.00	0.26	0.797	[-0.03, 0.04]	0.000					
	BMI	-0.03	-0.85	0.398	[-0.08, 0.03]	0.003					
	Ethnicity: Asian	-1.09	-4.76	**< 0.001**	[-1.51, -0.67]	0.079					
	Alexithymia	0.00	0.02	0.984	[-0.02, 0.02]	0.000					
	Time: async × ED risk: high	-0.21	-1.18	0.239	[-0.56, 0.14]	0.001					
Total	Time: async	-0.32	-2.80	**0.006**	[-0.43, -0.08]	0.006	1.06	0.50	0.71	0.10	0.68
	ED risk: high	0.13	0.72	0.472	[-0.21, 0.48]	0.001					
	Age	-0.03	-2.12	**0.036**	[-0.05, -0.00]	0.017					
	BMI	-0.02	-1.03	0.305	[-0.06, 0.02]	0.004					
	Ethnicity: Asian	-0.74	-4.64	**< 0.001**	[-1.05, -0.45]	0.075					
	Alexithymia	0.01	0.96	0.337	[-0.01, 0.02]	0.003					
	Time: async × ED risk: high	-0.09	-0.69	0.489	[-0.35, 0.16]	0.000					
Objective enfacement	Time: sync	-5.71	-5.99	**< 0.001**	[-7.47, -4.00]	0.012	187.26	55.82	0.78	0.03	0.77
	Time: async	-4.64	-4.87	**< 0.001**	[-6.52, -2.71]	0.008					
	ED risk: high	0.14	0.06	0.949	[-4.06, 4.71]	0.000					
	Age	-0.01	-0.07	0.941	[-0.33, 0.31]	0.000					
	BMI	0.11	0.41	0.686	[-0.42, 0.61]	0.001					
	Ethnicity: Asian	3.31	1.65	0.100	[-0.38, 7.00]	0.010					
	Alexithymia	0.01	0.11	0.914	[-0.18, 0.18]	0.000					
	Time: baseline × ED risk: high	1.50	1.06	0.291	[-1.11, 4.31]	0.000					
	Time: async × ED risk: high	0.02	0.01	0.988	[-2.91, 2.82]	0.000					
Facial attractiveness	Time: sync	0.06	0.99	0.325	[-0.06, 0.20]	0.000	1.63	0.27	0.88	0.11	0.86
	Time: async	-0.07	-1.11	0.268	[-0.19, 0.05]	0.000					
	ED risk: high	-0.53	-2.72	**0.007**	[-0.92, -0.15]	0.012					
	Age	-0.01	-0.54	0.590	[-0.04, 0.02]	0.001					
	BMI	0.00	0.13	0.899	[-0.04, 0.05]	0.000					
	Ethnicity: Asian	0.36	1.99	**0.047**	[0.03, 0.72]	0.016					
	Alexithymia	-0.02	-2.72	**0.007**	[-0.04, -0.01]	0.029					
	Time: sync × ED risk: high	-0.07	-0.76	0.446	[-0.27, 0.12]	0.000					
	Time: async × ED risk: high	-0.02	-0.16	0.872	[-0.21, 0.16]	0.000					
Facial adiposity	Time: sync	-0.02	-0.47	0.640	[-0.13, 0.07]	0.000	0.64	0.17	0.82	0.12	0.79
	Time: async	-0.02	-0.31	0.755	[-0.11, 0.09]	0.000					
	ED risk: high	0.28	2.20	**0.029**	[0.03, 0.53]	0.008					
	Age	0.01	0.52	0.605	[-0.01, 0.02]	0.001					
	BMI	0.04	2.58	**0.011**	[0.01, 0.07]	0.025					
	Ethnicity: Asian	-0.13	-1.12	0.264	[-0.37, 0.10]	0.005					
	Alexithymia	0.01	2.95	**0.004**	[0.01, 0.02]	0.032					
	Time: sync × ED risk: high	-0.01	-0.08	0.936	[-0.16, 0.15]	0.000					
	Time: async × ED risk: high	0.02	0.32	0.746	[-0.13, 0.18]	0.000					
Head dissatisfaction	Time: sync	-0.69	-1.81	0.070	[-1.38, 0.04]	0.000	77.98	8.85	0.92	0.20	0.90
	Time: async	-0.42	-1.11	0.268	[-1.05, 0.27]	0.000					
	ED risk: high	3.96	2.98	0.**003**	[1.54, 6.45]	0.014					
	Age	-0.07	-0.64	0.522	[-0.26, 0.14]	0.002					
	BMI	0.08	0.47	0.637	[-0.27, 0.38]	0.001					
	Ethnicity: Asian	0.67	0.54	0.593	[-1.74, 3.22]	0.001					
	Alexithymia	0.26	4.76	**< 0.001**	[0.16, 0.36]	0.085					
	Time: sync × ED risk: high	2.16	3.83	**< 0.001**	[1.09, 3.27]	0.002					
	Time: async × ED risk: high	2.03	3.60	**< 0.001**	[0.90, 3.10]	0.002					
Body dissatisfaction	Time: sync	-0.81	-2.59	**0.010**	[-1.37, -0.15]	0.001	80.65	6.02	0.95	0.24	0.93
	Time: async	-0.68	-2.17	**0.030**	[-1.24, -0.11]	0.000					
	ED risk: high	6.10	4.59	**< 0.001**	[3.43, 8.64]	0.033					
	Age	-0.10	-1.00	0.321	[-0.32, 0.12]	0.004					
	BMI	0.41	2.47	**0.014**	[0.07, 0.72]	0.025					
	Ethnicity: Asian	-1.45	-1.15	0.252	[-3.90, 1.02]	0.006					
	Alexithymia	0.21	3.89	**< 0.001**	[0.10, 0.33]	0.060					
	Time: sync × ED risk: high	1.79	3.85	**< 0.001**	[0.81, 2.64]	0.002					
	Time: async × ED risk: high	1.41	3.05	**0.002**	[0.58, 2.28]	0.001					
Dysmorphic concern	Time: sync	-1.54	-3.66	**< 0.001**	[-2.39, -0.72]	0.002	76.73	11.01	0.91	0.29	0.87
	Time: async	-0.91	-2.16	**0.031**	[-1.75, -0.09]	0.001					
	ED risk: high	9.12	6.83	**< 0.001**	[6.42, 11.72]	0.069					
	Age	-0.20	-1.98	**0.049**	[-0.40, -0.01]	0.016					
	BMI	0.10	0.60	0.552	[-0.22, 0.40]	0.001					
	Ethnicity: Asian	-4.07	-3.27	**0.001**	[-6.48, -1.77]	0.042					
	Alexithymia	0.15	2.73	**0.007**	[0.05, 0.25]	0.029					
	Time: sync × ED risk: high	1.54	2.46	**0.015**	[0.31, 2.76]	0.001					
	Time: async × ED risk: high	0.67	1.06	0.289	[-0.61, 1.84]	0.000					

*BMI* body mass index, *ED* Eating disorder, *CI* confidence interval (derived from bootstrapping), *LB* lower bound, *UB* upper bound, *b* regression coefficient, *sync* synchronous, async asynchronous, *ICC* intraclass correlation coefficient

Reference categories for time, ED risk, and ethnicity were assigned a value of 0; while categories shown in the table were assigned a value of 1. Significant *p* values (< 0.05) bolded

**Table 4:** Estimated marginal means and Post-Hoc pairwise comparisons between timing conditions per eating disorder risk group

Outcome Variable	Eating Disorder Risk Group	Estimated Marginal Means (EMMs)		Pairwise Comparison			
		Timepoint	EMM (*SE*)	Time Comparison	*MD*	*t*	*p*
Head dissatisfaction	Low	Baseline	22.7 (0.87)	Baseline - sync	0.69	1.81	0.211
		Sync	22.0 (0.87)	Baseline - async	0.42	1.11	0.803
		Async	22.3 (0.87)				
	High	Baseline	26.7 (0.97)	Baseline - sync	-1.47	-3.53	**0.001**
		Sync	28.2 (0.97)	Baseline - async	-1.61	-3.86	**< 0.001**
		Async	28.3 (0.97)				
Body dissatisfaction	Low	Baseline	24.0 (0.87)	Baseline - sync	0.81	2.59	**0.030**
		Sync	23.2 (0.87)	Baseline - async	0.68	2.18	0.091
		Async	23.3 (0.87)				
	High	Baseline	30.1 (0.97)	Baseline - sync	-0.98	-2.86	**0.014**
		Sync	31.1 (0.97)	Baseline - async	-0.74	-2.14	0.099
		Async	30.8 (0.97)				
Dysmorphic concern	Low	Baseline	32.0 (0.88)	Baseline - sync	1.54	3.66	**0.001**
		Sync	30.5 (0.88)	Baseline - async	0.91	2.16	0.093
		Async	31.1 (0.88)				
	High	Baseline	41.2 (0.97)	Baseline - sync	0.00	0.00	0.001
		Sync	41.2 (0.97)	Baseline - async	0.25	0.53	0.999
		Async	40.9 (0.97)				

*Sync* synchronous, *async* asynchronous, *SE* standard error, *MD* mean difference, *t* t-test. Significant *p* values (< 0.05) bolded

#### Objective enfacement

A significant main effect of Time was found for the self-face recognition task. Relative to baseline, participants required less self-content before identifying the morph as "self" after synchronous IMS (*M* reduction = 5.03%), indicating incorporation of the other's facial features into their self-face representation (*R*² ≈ 1%). A similar reduction was also observed following asynchronous IMS relative to baseline (*M* reduction = 4.63%; *R*² ≈ 1%). Synchronous and asynchronous conditions did not significantly differ when compared (see Supplementary Table S2). There was no significant main effect of Group nor any Time × Group interactions, indicating comparable shifts in self-recognition thresholds across ED-risk levels. The model explained 78% of the total variance (3% unique).

#### Subjective enfacement

A significant main effect of Time was observed for both the enfacement questionnaire total score and the self-identification subscale score, with participants reporting higher enfacement following synchronous compared to asynchronous IMS (*R*² ≈ 1–2%). Average scores fell below the scale’s affirmative threshold in both IMS conditions. No significant Time effects were found for the similarity or affect subscales. There were no significant effects of Group nor any Time × Group interactions, indicating comparable enfacement across ED-risk levels. Age and ethnicity were significant covariates, with younger participants and those identifying as White reporting higher enfacement questionnaire scores. The models explained 65–74% of the total variance (8–10% unique).

### Changes in face and body image disturbance post-enfacement illusion

On a descriptive level, high ED-risk participants showed greater face and body image disturbance at baseline (scores typically towards the scale extremes) compared to low ED-risk participants (scores typically around or below scale midpoints).

#### Facial attractiveness and adiposity

No significant main effects of Time (baseline vs. synchronous; baseline vs. asynchronous) nor any Time × Group interactions emerged, suggesting that perceived facial attractiveness and adiposity did not change following either IMS condition compared to baseline. The main effects of Group were significant, with high (relative to low) ED risk predicting lower perceived facial attractiveness and higher perceived facial adiposity across conditions (*R*² ≈ 1%). The models explained 82–88% of the total variance (11–12% unique).

#### Head dissatisfaction, body dissatisfaction, and dysmorphic concern

The main effect of Time was significant for body dissatisfaction and dysmorphic concern, with reduced disturbance following both synchronous and asynchronous IMS conditions compared to baseline (*R*² ≈ 0–1%). The main effect of Time for head dissatisfaction was non-significant, indicating no change from pre- to post-IMS. The main effects of Group were significant, with high (relative to low) ED risk predicting greater head dissatisfaction, body dissatisfaction, and dysmorphic concern across conditions (*R*² ≈ 1–7%).

Significant Time (synchronous vs. baseline) × Group interactions emerged for head dissatisfaction, body dissatisfaction, and dysmorphic concern (*R*² ≈ 0%). Additionally, significant Time (asynchronous vs. baseline) × Group interactions were found for head and body dissatisfaction (*R*² ≈ 0%). Post-hoc analyses revealed that high ED-risk participants showed increased head dissatisfaction following both synchronous and asynchronous IMS compared to baseline, as well as increased body dissatisfaction following synchronous IMS compared to baseline, but no change following asynchronous IMS. In contrast, low ED-risk participants showed decreased body dissatisfaction and dysmorphic concern following synchronous IMS compared to baseline, with no significant changes following asynchronous IMS.

Significant covariates emerged in some models, with younger age, higher BMI, Asian ethnicity, and greater alexithymia predicting more face and/or body image disturbance across conditions. The models explained 91–95% of the total variance (20–29% unique).

### Exploratory analyses: facial model effects

Model identity (i.e., which of the 10 models participants viewed) did not predict subjective or objective enfacement outcomes, and accounted for only minimal variance in face and body image outcomes (*R*² = 1–2%), without altering the main Time or Time × Group interaction effects (Supplementary Table S3). Similarly, model validation status (current vs. previous validation) was not a significant predictor in any of the models and did not change the primary pattern of findings (results are available upon request from the corresponding author). Perceived model attractiveness showed small, group-specific associations: lower attractiveness ratings were associated with greater body dissatisfaction among high ED-risk participants, but reduced dysmorphic concern among low ED-risk participants following synchronous IMS compared to baseline (Supplementary Tables S4–S5).

## Discussion

The current study investigated whether individuals at high versus low ED risk show differential susceptibility to a face-based multisensory illusion—the enfacement illusion—and whether experiencing the illusion can reduce face and body image disturbance. Contrary to expectations, participants at high versus low ED risk showed comparable susceptibility to the enfacement illusion across subjective and objective outcome measures. Partially consistent with expectations, experiencing the enfacement illusion led to reductions in specific body image disturbance outcomes. However, the observed shifts in body image disturbance diverged across ED risk groups in unexpected ways: low-ED-risk participants showed improvements, whilst high-ED-risk participants showed slight worsening.

### Eating disorder risk and susceptibility to the enfacement illusion

At the subjective level, both ED risk groups self-reported greater identification with the other-face after synchronous IMS compared to asynchronous IMS, consistent with an enfacement effect (i.e., shifts in self-face representation were dependent on temporal synchrony between visual and motor inputs—consistent with multisensory integration mechanisms). However, the observed subjective enfacement effect was modest, since average self-reported scores fell below the scale’s affirmative threshold across conditions—a finding that is consistent with general observations in the enfacement literature (e.g [45, 47, 78]) for a review, see [83]. Many researchers suggest that shifts in self-face representation are difficult to induce, at least more so than shifts in bodily-self representation, possibly due to the face’s intrinsic connection to identity [45, 83]. Importantly, the absence of differences in enfacement illusion susceptibility across ED risk groups suggests that basic multisensory (visuo-motor) integration processes underlying self-face representation and its plasticity, and supporting the integrity of self-other boundaries, may not be impaired in individuals with ED vulnerability.

These findings broadly differ from previous embodiment illusion research, where rubber hand illusion strength is typically enhanced in both clinical ED patients (current and recovered) compared to healthy controls [30, 84, 85], and in community-based samples with elevated ED symptomatology [85–87], with similar medium to large effects [27]. These previous results suggest that altered multisensory integration processes may underpin distorted bodily self-perception in EDs. Within a predictive coding framework, heightened susceptibility to bodily illusions in EDs has been attributed to an over-reliance on rigid, distorted body priors (beliefs) and an under-reliance on current sensory input—especially when the input is ambiguous or imprecise—leading to erroneous, externally-driven updates in bodily self-representation [88–90].

However, the current findings suggest that this predictive coding account may not generalize across body regions. Unlike the hand, which is affectively neutral, the face holds affective salience in EDs [41]. Studies involving full-body illusions targeting the abdomen, another affectively salient region in EDs, have similarly yielded non-significant or attenuated ED-related effects on illusion susceptibility (e.g [31, 91]), whereas hand-based embodiment illusions consistently show ED-related effects [27]. Emotionally- or identity-salient body regions may be governed by qualitatively different or stronger top-down priors than more neutral body regions. In the facial domain, the centrality of the face to self-identity and social functioning [92] may constrain multisensory plasticity. This interpretation positions our null finding as theoretically meaningful: EDs may not be associated with a pathological disruption in the fundamental multisensory integration processes governing face perception, possibly because the face serves broader identity and social functions.

At the objective level, ED-risk groups also showed comparable shifts in self-face recognition thresholds during the self-other morphing task. Participants required fewer self-features to recognise a face as “self” following both synchronous (5.03% reduction) and asynchronous (4.63% reduction) IMS compared to baseline, indicating increased assimilation of the other's face into one's self-representation. Although the shift was numerically larger following synchronous IMS, differences between synchronous and asynchronous conditions were not significant. This pattern is consistent with prior enfacement studies reporting baseline-to-post IMS shifts in both conditions (e.g [70, 77] but see [78, 83]. These findings suggest that mechanisms beyond multisensory (visuo-motor) integration may contribute to objective changes in self-face representation (i.e., self-other boundaries). One possibility is affective resonance or emotional contagion during facial mimicry. Prior research suggests that mimicking or simply attending to the emotional expressions of another person’s face can elicit affective resonance and perceived self-other overlap [55]. Although our affect subscale of the enfacement questionnaire showed no significant difference between synchronous and asynchronous IMS conditions, scores were numerically closer to the neutral midpoint in the synchronous condition (*M* = -0.11) than in the other subscales (self-identification and similarity; *M* = -0.70 to -0.73), suggesting modest emotional contagion may have occurred. Notably, neither IMS condition differentiated ED-risk groups, suggesting that the perceptual processes supporting self-face representation and its plasticity may operate independently of ED symptom severity.

We also acknowledge that while our objective measure (self-face recognition task) produced comparable effects to classic studies involving tactile stroking methods (e.g [46]), the modest subjective effects and absence of ED-related differences may reflect limitations of the current paradigm. The use of facial mimicry rather than tactile stroking [83], or passive (experimenter-controlled) rather than active (participant-controlled) movements—which the limited research involving facial mimicry has supported [55]—may have attenuated enfacement strength: ultimately, obscuring possible ED-related differences. However, existing evidence on the relative efficacy of these stimulation types remains sparse, and there is currently no robust evidence that visuo-tactile or active paradigms are most efficacious. Moreover, because the face (like the hand) has high tactile receptor density in the brain [93], some tactile contribution may still occur during facial mimicry. Future research should systematically compare stimulation modalities and movement types to determine optimal parameters for enfacement induction in ED-related populations.

### Changes in body image following the enfacement illusion

Partially consistent with expectations, experiencing the enfacement illusion led to modest reductions in body image disturbance across the entire sample: body dissatisfaction and dysmorphic concern reduced following both synchronous and asynchronous IMS compared to baseline. These effects broadly align with full-body illusion research demonstrating reductions in body dissatisfaction after embodying an external body in community samples (e.g [35] for a review, see [27]). However, we failed to observe changes in face-specific body image, namely, head dissatisfaction, perceived facial attractiveness or adiposity.

Shifts in body image diverged across ED risk groups in unexpected ways. While low ED-risk participants reported reduced body dissatisfaction and dysmorphic concern following synchronous IMS compared to baseline, high ED-risk participants reported slight increases in head and body dissatisfaction following both synchronous and asynchronous IMS conditions compared to baseline. Broadly, these findings differ from full-body illusion studies showing that improvements in body image following the illusion were most pronounced among individuals with elevated ED symptoms, both in community samples (e.g [35]) and in ED patients compared to healthy controls (e.g [31, 84] for a review, see [27]).

Predictive coding theory provides a useful interpretive framework. Embodiment illusion interventions are theorised to generate improvements in body image by inducing prediction errors that prompt updating of the internal body model (body representation) in line with the current sensory input, provided the input is granted sufficient precision [28, 89]. Such updating induces subsequent shifts in higher-level beliefs and attitudes about the body [34]. In full-body illusion studies, individuals with elevated ED symptom levels often show stronger responses [27], presumably because their internal body representations are distorted, rigid, or maintained by over-precise high-level priors (e.g., “I am unattractive”) [28, 90]. When a salient and reliable discrepancy (prediction error) is introduced (e.g., embodying a slimmer or average-sized body), the incoming sensory input drives adaptive perceptual updating [25, 82]. If, instead, the incoming sensory input is perceived as unreliable or even aversive, prediction errors may be dismissed [89].

High-ED-risk individuals typically hold rigid, negatively biased beliefs (priors) about their appearance; thus, for them, the sensory input (Other face) may have been encoded as unreliable or aversive, reducing the precision of the prediction error and instead, reinforcing rather than challenging negative priors. Supporting this interpretation, exploratory analyses showed that lower perceived attractiveness of the model was linked to increased body dissatisfaction in high-ED-risk individuals, but reduced dysmorphic concern in low-ED-risk participants. This pattern is consistent with prior evidence from full-body illusion studies showing that individuals with ED symptomatology often evaluate healthy-weight or average bodies (i.e., consistent with our facial models of ‘average’ adiposity) as unattractive, even when recognising them as similar to themselves [94]. Therefore, for high-ED-risk participants, experiencing the enfacement illusion with another face perceived as less attractive may have contributed to the observed negative shifts in body image.

 However, the negative shifts observed in the high-ED-risk group following both synchronous and asynchronous IMS conditions suggest that these effects were unlikely to be driven by multisensory integration alone. This may reflect mere visual exposure to another’s face, or more complex socio-emotional factors. One possibility is that asynchronous IMS, which preserves self-other distinction more than synchronous IMS, maintains awareness of being observed and evaluated. Faces hold inherent social salience and are central to threat detection [95] and interpersonal evaluation (e.g., via shifts in gaze or emotional expression) [96]. Individuals with elevated ED symptoms often exhibit maladaptive social cognition, including heightened appearance-based rejection sensitivity, negative interpretation biases of ambiguous social cues [97], and attentional avoidance of others’ faces, especially emotionally expressive ones [98, 99]. Thus, although speculative, the enfacement illusion task may have been perceived as socially threatening, eliciting a schema-consistent interpretation of social cues (e.g., changes in emotional expression interpreted as evaluative or rejecting), which amplified head and body dissatisfaction. This interpretation aligns with predictive coding models of psychopathology [100], such as Badcock et al.’s [101] model of depressive mood, which proposes that psychopathology is a response to uncertain or threatening social contexts. Individuals with mental health conditions assign excessively high precision to social prediction errors (e.g., discrepancies between actual and preferred social outcomes), which, over time, strengthen negative priors (e.g., expecting negative social outcomes). Such expectations subsequently bias attention and the interpretation of sensory input in a schema-consistent manner [101]. Applied to the current study, high-ED-risk participants may have approached the enfacement illusion with strong appearance-related priors. The socially salient stimulus (an unfamiliar face gazing and changing expressions) may have heightened attention to possible interpersonal evaluation. Ambiguous or neutral facial cues (e.g., simple changes in the model’s facial expression) could then be interpreted through a negative schema (e.g., “I am unattractive,” “Others see me this way”), producing the observed increase in head and body dissatisfaction.

The Allocentric Lock Theory [102] offers a complementary neuroscientific account. This theory suggests that individuals with EDs are “locked” into maladaptive, observer-based (allocentric) body representations that resist updating from real-time, first-person (egocentric) sensory input. In the context of the enfacement illusion, if all participants, but importantly high ED-risk participants, maintained a sense of perceptual self–other boundaries, this may have limited the degree of egocentric updating—possibly reinforcing internalised, critical self-evaluations from an allocentric perspective.

 Finally, we note that the specific changes in head dissatisfaction, without corresponding changes in perceptions of facial attractiveness or facial adiposity, may reflect measurement sensitivity differences. The multi-item composite measure of head dissatisfaction may capture feature-specific concerns that align with documented attentional biases toward specific facial features in ED populations [103]. In contrast, the single-item measures of facial attractiveness and adiposity may lack the sensitivity to detect subtle changes in evaluative concerns in high ED-risk participants. Face-specific body image disturbance may also be more ingrained and resistant to change in EDs due to the face’s connection to identity, even when targeted by enfacement illusions. This is further supported by stable facial attractiveness and adiposity ratings across groups, as well as the slightly greater variance explained by the body dissatisfaction model (24%) compared to the head dissatisfaction model (20%).

### Strengths, limitations, and future directions

Study strengths include a robust and sufficiently powered sample size across both ED-risk groups, comprehensive outcome measures spanning face and body evaluations, control for several potential confounders (i.e., age, BMI, ethnicity, and alexithymia), and controlled comparisons between synchronous and asynchronous conditions, enhancing the robustness of the observed effects. 

Nonetheless, several unaddressed methodological limitations warrant consideration. First, while our novel online procedure produced comparable subjective effects [83] and objective metrics to lab-based studies [46], uncontrolled environmental variability (e.g., lighting or screen size) may have attenuated effects. Replication in controlled laboratory settings would help to confirm the reliability of the current findings.

Second, the facial stimuli (i.e., models) used in the enfacement paradigm were partially adapted from prior work without complete pilot validation data for all models. Although supplementary analyses showed that model identity explained minimal variance (≤ 2%) without altering core findings, the absence of systematic stimulus manipulation remains a limitation. Additionally, facial models did not systematically vary in appearance, despite evidence that such variation influences therapeutic outcomes in ED populations [49, 50]. Future research should ensure comprehensive validation of all stimuli and consider systematically manipulating model attributes (e.g., tailored to participants’ appearance ideals) to enhance therapeutic potential.

Third, although the BSS and BICI-10 generally showed excellent internal consistency in the present sample, neither scale has been formally validated for state-based assessment. Hence, the observed body image changes should be interpreted cautiously. Future research should employ measures specifically validated for state assessment, such as the Body Image States Scale (BISS) [104], which has demonstrated sensitivity to embodiment illusion-based manipulations [34, 104]. Alternatively, researchers could supplement adapted measures with single-item visual analogue scales (e.g., “How dissatisfied are you with your appearance right now?“) to triangulate findings.

Fourth, the 5-minute interval between synchronous and asynchronous IMS conditions may have introduced carryover effects. This brief interval aligns with previous embodiment and enfacement studies that successfully identified synchronous versus asynchronous condition differences (e.g [62]), other studies use longer intervals (e.g., 1-hour or 1 day [78, 80]) or between-subjects designs, though systematic comparisons are lacking. Moreover, we prioritised environmental consistency in our remote (online) procedure, since longer breaks would have increased uncontrolled variability (e.g., lighting, positioning, distractions). Nonetheless, carryover may have attenuated condition differences or contributed to some null findings. Future research should systematically compare inter-condition intervals and within versus between-subjects designs to determine optimal procedures.

Fifth, although our sample varied in ED risk, a clinically diagnosed ED sample may increase sensitivity to detect differences in susceptibility. Indeed, prior research has more commonly reported differences in rubber hand illusion susceptibility based on clinical ED diagnosis (versus controls) than based on levels of ED symptoms in community samples: however, similar medium to large effects were found across findings [27]. Additionally, greater heterogeneity within the high-ED-risk group in terms of ED symptom levels (i.e., larger standard deviations regarding EAT-26 scores) compared to the low-ED-risk group may have reduced sensitivity to detect group differences. Future studies should consider including formally diagnosed ED patients to clarify whether the observed findings generalise to clinical contexts.

Finally, the current study did not assess comorbid affective or neurodevelopmental traits or conditions (e.g., depression, anxiety, autistic spectrum traits) among populations with ED symptomatology, despite their known influence on bodily-self-representation and embodiment illusion susceptibility [88]. In particular, dispositional empathetic responding has been shown to increase enfacement susceptibility [45]. These factors could moderate responses to self-face updating, particularly given the social nature of the manipulation. Future enfacement research could assess the impact of affect-related traits and states on illusion strength and therapeutic responsiveness in ED populations.

Additionally, to clarify the mechanisms underlying susceptibility and body image changes, future enfacement studies should consider stricter experimental control conditions. For example, passive viewing without motor engagement (e.g., viewing static faces with neutral or smiling expressions) [55] or isolated motor tasks (e.g., performing facial movements without viewing another face) could help to isolate the effects of multisensory integration from other factors (e.g., mere exposure, emotional contagion). Combining the enfacement paradigm with facial electromyographic recording could also help to disentangle multisensory integration effects from emotional contagion. For understanding body image changes specifically, additional controls that manipulate the socio-evaluative context (e.g., neutral vs. smiling expressions, gaze direction, non-social comparison stimuli) may be useful. Future studies could also assess the role of socio-emotional processing biases (e.g., rejection sensitivity), alongside the aforementioned affective traits, in modulating affective responses to enfacement in ED-vulnerable individuals. 

### Implications and conclusions

While the current findings pertain to a high-ED-risk sample, which may not generalise uniformly to a clinical ED sample, they offer new insight into the nature of bodily self-perception in EDs. The observation that susceptibility to the enfacement illusion—both subjectively and objectively—did not differ across ED risk groups suggests that the multisensory processes supporting self-face recognition and self-other face discrimination may not be impaired at elevated ED risk levels. This finding may help to refine theories of altered multisensory integration and exteroceptive processing bias underlying distortions in bodily self-perception in EDs [28], suggesting that such abnormalities may not similarly extend to self-face perception. However, a dissociation emerged between the perceptual mechanism and its evaluative outcome: although both groups similarly updated their self-face representations, only high ED-risk participants showed negative shifts in body image following the illusion.

These findings may refine and complement emerging theories of bodily identity in EDs [96]. Bodily identity has been defined as the integrated, perceptual sense of one’s body as uniquely “mine,” shaped by the dynamic interplay between interoceptive bodily signals (inner body), exteroceptive perceptions of one’s physical appearance (outer body), and the social environment within which such perceptions are embedded (social body) [17, 34]. This form of identity is considered to be grounded in bottom-up multisensory integration rather than top-down cognition [17, 18] and is distinct from narrative identity—the abstract, evaluative self-concept shaped by memory, values, and cognitive appraisals [96]. Enfacement illusions are an important tool for probing bodily identity by altering not only self-representations but social representations of the self, through the blurring of perceptual self-other boundaries [17, 26, 47].

While EDs may not necessarily involve a fundamental erosion of bodily identity—at least not within socially embedded contexts like self–other facial discrimination and processing—narrative identity (the evaluative, cognitive-affective layer) may remain dysfunctional [96]. In this sense, in ED populations, the face may be stably represented at a lower-order, perceptual level reflecting the integration of internal and external sensory experiences, despite being perceived as unacceptable or flawed at a higher-order, evaluative level. Such higher-order disturbances may arise within the context of social or aesthetic standards imposed externally—reflecting what Riva [34] terms the objectified (social) body.

Clinically, in this view, pending replication, ED interventions aimed at recalibrating affective and cognitive components of body image (e.g., body dissatisfaction, internalisation of beauty ideals) may be more appropriate than those targeting deeper disruptions in face-specific bodily identity or self-other processing.

Additionally, the current findings suggest that enfacement-based interventions may offer limited clinical benefit for reducing face or body image disturbance among individuals with or at high risk of EDs. One possibility is that, due to maladaptive social cognition processes, high ED-risk individuals interpreted the enfacement illusion as a socially evaluative encounter, reinforcing self-critical schemas and/or a negative observer-based perspective of the self. In effect, the illusion became a socially charged mirror, reinforcing maladaptive schemas rather than updating them. While this interpretation is speculative, it nonetheless prompts caution when incorporating facial stimuli into embodiment-based interventions, particularly without accounting for individual differences in social cognition that may shape how such stimuli are perceived and internalised.

As embodiment-based interventions continue to gain traction in ED treatment research [27, 105], future work should move beyond purely perceptual models to consider the critical roles of social cognition, affective processing, and identity relevance. Integrating these dimensions may enhance the efficacy of such interventions and better address the complex, multi-layered nature of body image disturbance in ED populations.

Ultimately, while the enfacement illusion shows promise as a research tool for better understanding multisensory contributions to body image, considerably more investigation is warranted before clinical implementation can be responsibly recommended.

## Supplementary Information

Below is the link to the electronic supplementary material.

Supplementary Material 1.

## Data Availability

The datasets used and/or analysed during the current study are available from the corresponding author on reasonable request.
