# Supplementary material for: Rethinking the bodily self: evidence from the enfacement illusion in women at risk for eating disorders
Source: J Eat Disord. 2025 Dec 23;14:29. doi: 10.1186/s40337-025-01461-1 (PMC12837083; doi:10.1186/s40337-025-01461-1)
Supplement: Supplementary file 1 — Supplementary Material 1. [file 40337_2025_1461_MOESM1_ESM.docx]

**Supplementary Material**

**Supplementary Figure S1**

*Example Model Images Used in Simulation Videos and Self-Face Recognition Task*


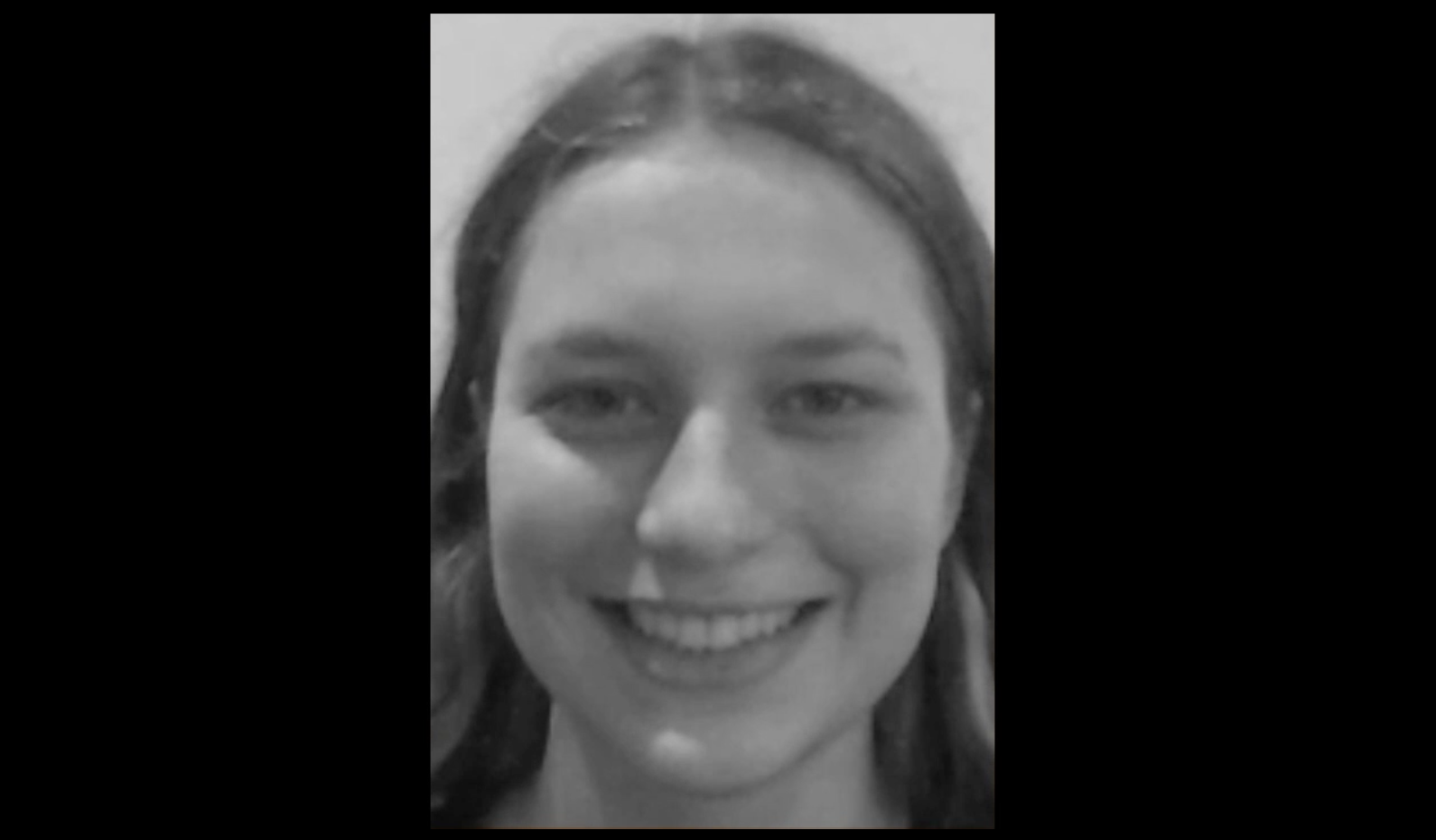

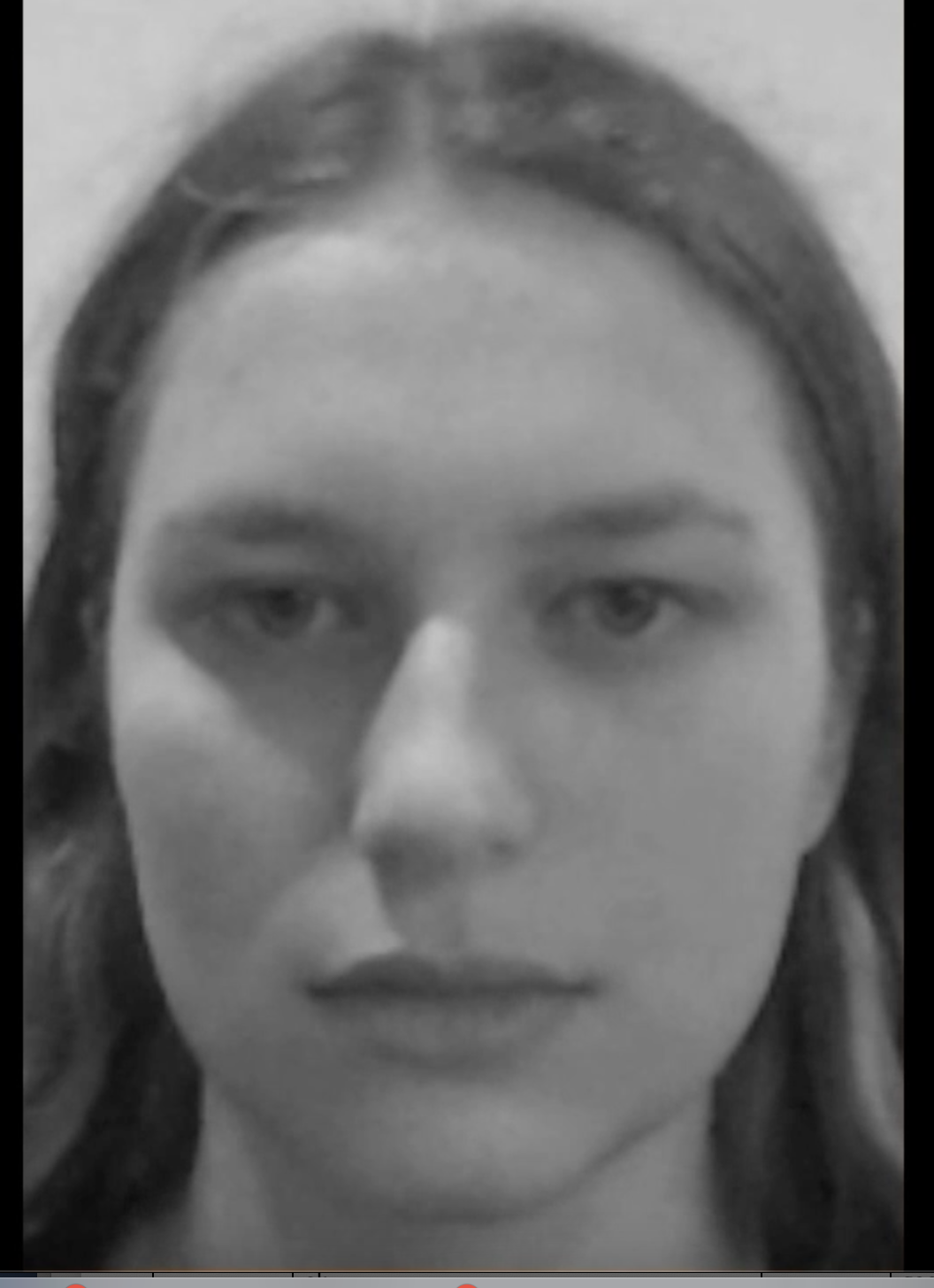


**
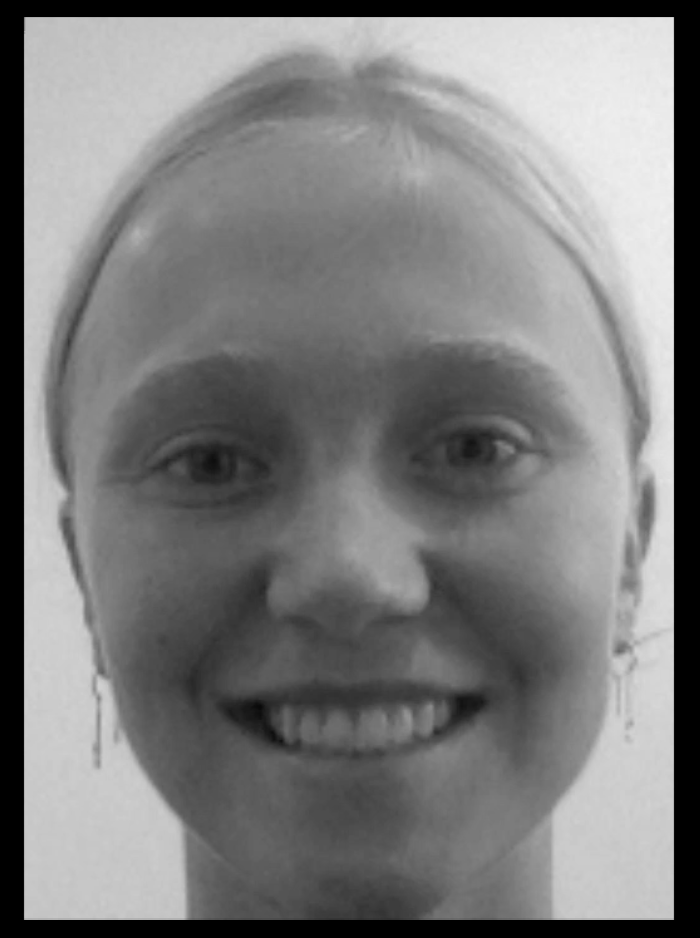

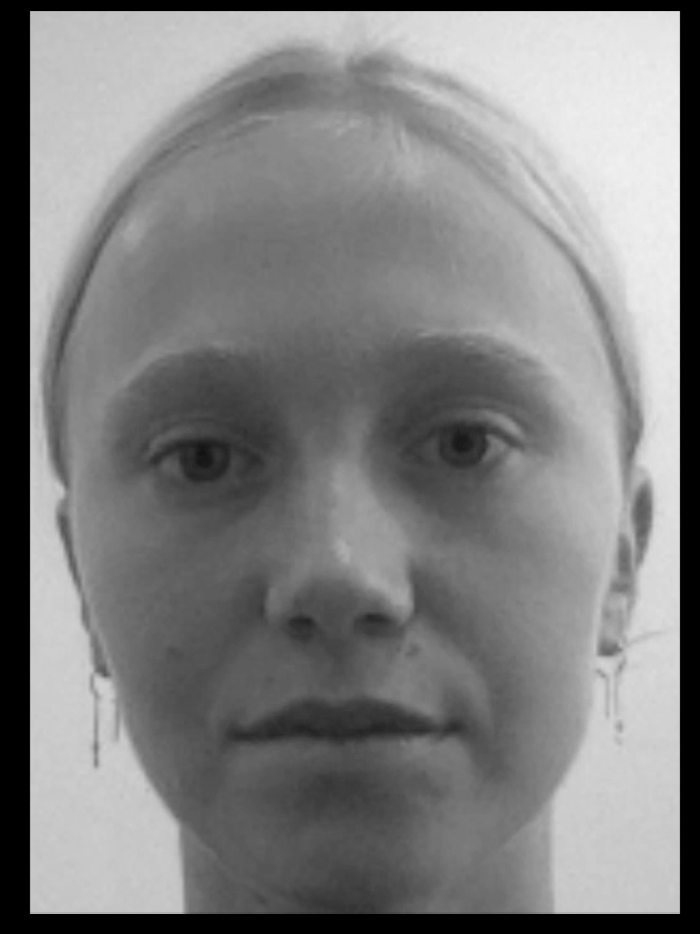
**

**
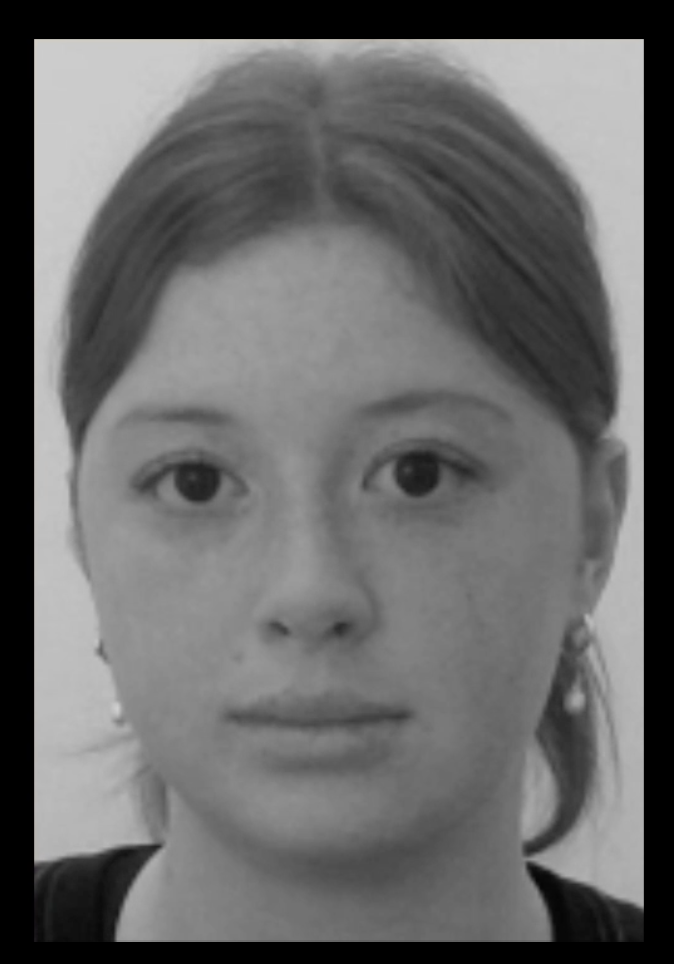

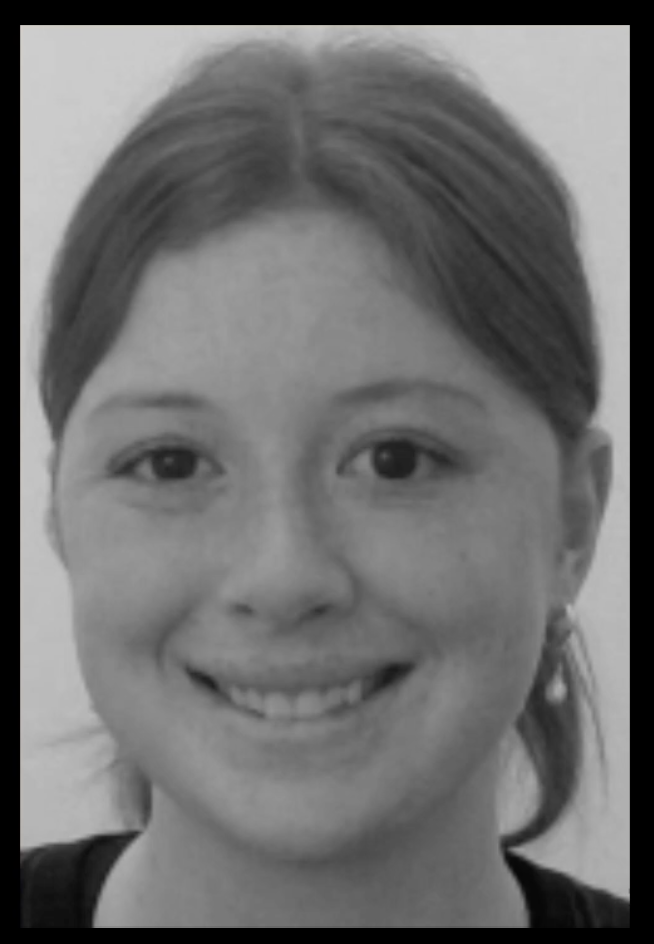
**

*Note.* Depicted are examples of three models of White ethnicity. Left images show neutral expression, right images show smiling expression.

**Table S1**

*Pilot Study Model Ratings: Descriptive Statistics*

| Model Ethnicity | Attractiveness | | | | Adiposity | | | | Likeability | | | | |
| --- | --- | --- | --- | --- | --- | --- | --- | --- | --- | --- | --- | --- | --- |
|  | Min | Max | *M* | *SD* | Min | Max | *M* | *SD* | Min | Max | *M* | *SD* |  |
| White |  |  |  |  |  |  |  |  |  |  |  |  |  |
| W1 | -1 | 2 | .77 | 1.032 | -1 | 1 | -.12 | .431 | -1 | 2 | 1.31 | 1.011 |  |
| W2 | -2 | 3 | .92 | 1.498 | -1 | 0 | -.16 | .374 | -2 | 3 | .28 | 1.458 |  |
| W3 | -3 | 2 | .24 | 1.091 | -2 | 0 | -.16 | .473 | -2 | 2 | -.08 | 1.320 |  |
| W4 | -3 | 2 | -.40 | 1.080 | -1 | 1 | .04 | .351 | -2 | 2 | .08 | 1.256 |  |
| W5 * | -2 | 2 | 0.04 | 1.274 | 0 | 1 | .20 | .408 | -2 | 2 | .40 | 1.225 |  |
| W6 * | -2 | 2 | 0.46 | 1.062 | -1 | 0 | -.13 | .338 | -2 | 2 | -.38 | 1.056 |  |
| W7 | 0 | 3 | 1.33 | .868 | -1 | 0 | -.17 | .381 | -1 | 2 | .42 | 1.060 |  |
| W8 | -1 | 2 | .00 | .978 | 0 | 1 | .13 | .338 | -1 | 2 | 1.13 | .992 |  |
| W9 * | -2 | 2 | -.04 | 1.083 | -1 | 1 | .00 | .295 | -2 | 2 | .04 | 1.160 |  |
| W10 | -3 | 1 | -.33 | .868 | -2 | 0 | -.21 | .588 | -2 | 2 | -.08 | 1.139 |  |
| East/Southeast Asian |  |  |  |  |  |  |  |  |  |  |  |  |  |
| W1 | -1 | 3 | .81 | 1.009 | -2 | 1 | -.17 | .507 | -2 | 3 | 1.22 | 1.267 |  |
| W2 * | -2 | 3 | -.20 | 1.183 | -2 | 1 | -.23 | .547 | -2 | 3 | .46 | 1.268 |  |
| W3 | -2 | 3 | .54 | 1.197 | -3 | 1 | -.46 | .852 | -2 | 3 | .60 | 1.288 |  |
| W4 | -2 | 3 | .43 | 1.145 | -2 | 2 | .49 | .781 | -2 | 3 | .63 | 1.308 |  |
| W5 * | -2 | 3 | .00 | 1.372 | -3 | 0 | -.40 | .736 | -2 | 3 | .20 | 1.302 |  |
| W6 | -3 | 3 | -.34 | 1.327 | -3 | 0 | -1.03 | .985 | -2 | 3 | .06 | 1.235 |  |
| W7 | -2 | 3 | -.51 | 1.358 | -3 | 2 | -.17 | .985 | -3 | 3 | .14 | 1.396 |  |
| W8 | -2 | 3 | .11 | 1.323 | -3 | 0 | -.51 | .781 | -2 | 3 | .34 | 1.327 |  |
| W9 * | -2 | 3 | .26 | 1.314 | -2 | 3 | -.06 | .873 | -2 | 3 | .37 | 1.330 |  |
| W10 | -3 | 3 | -.71 | 1.382 | -1 | 2 | .53 | .615 | -2 | 3 | .53 | 1.376 |  |

Note. Pilot data from the current study for 6 models (3 White, 3 East/Southeast Asian) selected for the main experiment. N raters: 24 (White), 35 (East/Southeast Asian). Models marked with * were used in the main study. Ratings used 7-point Likert scales: attractiveness (-3 = very unattractive, +3 = very attractive), adiposity (-3 = very underweight, +3 = very overweight), likeability (-3 = very unlikeable, +3 = very likeable). Four additional models (3 White, 1 East/Southeast Asian) were adopted from previous laboratory research; however, pilot data for these models are unavailable.

**Additional Main Analyses**

***Bootstrapping***

The bootstrapped 95% CIs largely confirmed the patterns observed in the main models. Significant main effects and interactions from the original models remained significant, with bootstrapped CIs excluding zero. Non-significant effects also remained non-significant, as indicated by CIs that included zero. One minor discrepancy was observed for age in a single model, where the original significance was not supported by the bootstrapped CI. This reflects the variability captured by bootstrapping. These results support the robustness of the original findings.

**Exploratory Analyses with Additional Covariates**

***Model Number***

To examine whether specific face models influenced outcomes, we re-analysed all hypotheses including model number as a covariate (1–6 for White participants; 1–4 for East/Southeast Asian participants). For H1 (enfacement susceptibility), model number was non-significant across all models and did not alter results. For Hypotheses 2–3 (body image outcomes), model number significantly predicted facial attractiveness ratings and head dissatisfaction across time-points. However, including this covariate did not change the significance of primary effects (time main effects for H2; time × ED risk interactions for H3). These analyses suggest model-specific variance was modest and did not substantially affect key findings. Full results are provided in Supplementary Table S3.

***Perceived Model Attractiveness and Desirability***

In addition to the primary models, we re-ran all analyses with an additional covariate: perceived desirability of the model (H1) and self-perceived model attractiveness (H2). **Perceived desirability of the model (H1)** was derived from Item 16 on the Discrete Emotions Questionnaire (DEQ; Harmon-Jones et al., 2016), which assesses emotional responses. Participants rated how desirable they found the model on a scale of 1 (*not at all*) to 7 (*extremely*). **Self-perceived model attractiveness (H2-H3)** was derived from Item 6 on the Enfacement Questionnaire (EQ; Panagiotopoulou et al., 2017). Participants rated how attractive they found the model on a scale of -3 (*not at all*) to 3 (*extremely*). Note, we did not control for perceived attractiveness in models testing H1, since the item was derived directly for the H1-outcome variable, so instead, included a separate but related construct.

Both covariates were derived from the mean score across synchronous and asynchronous conditions, addressing multicollinearity concerns arising from highly correlated predictors.

Tables S4 and S5 present detailed statistical results, including descriptive statistics, fixed effects, model fit, and post-hoc pairwise comparisons. The inclusion of these covariates did not alter the significance of main effects or interactions in any models. On average, higher perceived model desirability predicted greater enfacement questionnaire scores, while higher perceived model attractiveness predicted increased body dissatisfaction but reduced dysmorphic concern. We ran post-hoc pairwise comparisons on MLMs where the new covariate was significant. Post-hoc analyses revealed that when **controlling for higher** perceived model desirably (*M* = 1.95), both high and low ED risk participants showed greater subjective enfacement following synchronous versus asynchronous stimulation. Furthermore, when **controlling for lower perceived model attractiveness** (*M* = -0.39), **high ED risk participants** showed a **marginally significant increase** in body dissatisfaction following **synchronous stimulation** compared to baseline, while low ED risk participants showed a **significant decrease** in dysmorphic concern following **synchronous stimulation** compared to baseline.

**Table S2**

*Multilevel Modelling Results for Objective Enfacement Outcome with Baseline vs Synchronous and Synchronous vs Asynchronous Timing Contrasts*

| Outcome Variable | Fixed Effects | *b* | *t* | *p* | *b* Bootstrapped 95% CI (LB, UB) | Random Effects (Variance) | | Conditional *R*² | | Marginal *R*² | ICC |
| --- | --- | --- | --- | --- | --- | --- | --- | --- | --- | --- | --- |
|  |  |  |  |  |  | Intercept | Residual |  | |  |  |
| **Objective enfacement** | Time: baseline | 5.71 | 5.99 | **.001** | [3.69, 7.61] | 187.26 | 55.82 | .78 | | .03 | .77 |
|  | Time: async | 1.07 | 1.12 | .263 | [-0.88, 2.97] |  |  |  |  |  |  |
|  | ED risk: high | 1.64 | 0.74 | .460 | [-2.47, 6.19] |  |  |  | |  |  |
|  | Age | -0.01 | -0.07 | .941 | [-0.33, 0.31] |  |  |  | |  |  |
|  | BMI | 0.11 | 0.41 | .686 | [-0.38, 0.64] |  |  |  | |  |  |
|  | Ethnicity: Asian | 3.31 | 1.65 | .100 | [-0.55, 7.41] |  |  |  | |  |  |
|  | Alexithymia | 0.01 | 0.11 | .914 | [-0.16, 0.17] |  |  |  | |  |  |
|  | Time: baseline × ED risk: high | -1.50 | -1.06 | .291 | [-4.29, 1.28] |  |  |  | |  |  |
|  | Time: async × ED risk: high | -1.48 | -1.04 | .297 | [-4.07, 1.28] |  |  |  | |  |  |

*Note:* BMI = body mass index; ED = Eating disorder; CI = confidence interval (derived from bootstrapping); LB = lower bound; UB = upper bound; *b* = regression coefficient; sync = synchronous; async = asynchronous; ICC = intraclass correlation coefficient. Reference categories for time, ED risk, and ethnicity were assigned a value of 0; whilst categories shown in the table were assigned a value of 1. Significant *p* values (<.05) bolded.

**Table S3**

*Multilevel Modelling Results for Main Outcome Variables with Additional Covariate: Model Identity*

| Outcome variable | Fixed effects | *b* | *t* | *p* | Semi-partial *R*² | Random effects (variance) | | Conditional *R*² | Marginal *R*² |
| --- | --- | --- | --- | --- | --- | --- | --- | --- | --- |
|  |  |  |  |  |  | Intercept | Residual |  |  |
| Subjective enfacement (total score) |  |  |  |  |  | 1.07 | 0.50 | 0.71 | 0.10 |
|  | Time: sync | -0.25 | -2.81 | **0.005** | 0.01 |  |  |  |  |
|  | ED risk: high | 0.11 | 0.63 | 0.532 | 0.00 |  |  |  |  |
|  | Age | -0.03 | -2.12 | **0.036** | 0.02 |  |  |  |  |
|  | BMI | -0.02 | -1.00 | 0.320 | 0.00 |  |  |  |  |
|  | Ethnicity: Asian | -0.77 | -4.59 | **0.000** | 0.07 |  |  |  |  |
|  | Alexithymia | 0.01 | 1.04 | 0.301 | 0.00 |  |  |  |  |
|  | Model number | -0.03 | -0.52 | 0.606 | 0.00 |  |  |  |  |
|  | Time: sync × ED risk: high | -0.08 | -0.60 | 0.548 | 0.00 |  |  |  |  |
| **Objective enfacement** |  |  |  |  |  | 188.77 | 55.69 | 0.78 | 0.03 |
|  | Time: baseline | 5.71 | 6.00 | **<0.001** | 0.01 |  |  |  |  |
|  | Time: sync | 1.07 | 1.12 | 0.262 | 0.00 |  |  |  |  |
|  | ED risk: high | 1.93 | 0.86 | 0.390 | 0.00 |  |  |  |  |
|  | Age | -0.01 | -0.09 | 0.932 | 0.00 |  |  |  |  |
|  | BMI | 0.10 | 0.36 | 0.718 | 0.00 |  |  |  |  |
|  | Ethnicity: Asian | 3.68 | 1.75 | 0.082 | 0.01 |  |  |  |  |
|  | Alexithymia | 0.00 | 0.00 | 0.998 | 0.00 |  |  |  |  |
|  | Model number | 0.48 | 0.66 | 0.508 | 0.00 |  |  |  |  |
|  | Time: baseline × ED risk: high | -1.61 | -1.13 | 0.258 | 0.00 |  |  |  |  |
|  | Time: sync × ED risk: high | -1.66 | -1.17 | 0.243 | 0.00 |  |  |  |  |
| **Facial attractiveness** |  |  |  |  |  | 1.58 | 0.27 | 0.88 | 0.14 |
|  | Time: sync | 0.06 | 0.98 | 0.326 | 0.00 |  |  |  |  |
|  | Time: async | -0.07 | -1.11 | 0.269 | 0.00 |  |  |  |  |
|  | ED risk: high | -0.50 | -2.54 | **0.012** | 0.01 |  |  |  |  |
|  | Age | -0.01 | -0.45 | 0.651 | 0.00 |  |  |  |  |
|  | BMI | 0.00 | 0.05 | 0.957 | 0.00 |  |  |  |  |
|  | Ethnicity: Asian | 0.52 | 2.76 | **0.006** | 0.03 |  |  |  |  |
|  | Alexithymia | -0.03 | -3.16 | **0.002** | 0.04 |  |  |  |  |
|  | Model number | 0.16 | 2.41 | **0.017** | 0.02 |  |  |  |  |
|  | Time: sync × ED risk: high | -0.07 | -0.76 | 0.448 | 0.00 |  |  |  |  |
|  | Time: async × ED risk: high | -0.02 | -0.17 | 0.866 | 0.00 |  |  |  |  |
| **Facial adiposity** |  |  |  |  |  | 0.64 | 0.17 | 0.82 | 0.12 |
|  | Time: sync | -0.02 | -0.47 | 0.640 | 0.00 |  |  |  |  |
|  | Time: async | -0.02 | -0.31 | 0.755 | 0.00 |  |  |  |  |
|  | ED risk: high | 0.27 | 2.06 | **0.040** | 0.01 |  |  |  |  |
|  | Age | 0.01 | 0.52 | 0.601 | 0.00 |  |  |  |  |
|  | BMI | 0.04 | 2.61 | **0.010** | 0.03 |  |  |  |  |
|  | Ethnicity: Asian | -0.16 | -1.30 | 0.196 | 0.01 |  |  |  |  |
|  | Alexithymia | 0.02 | 3.03 | **0.003** | 0.03 |  |  |  |  |
|  | Model number | -0.03 | -0.83 | 0.407 | 0.00 |  |  |  |  |
|  | Time: sync × ED risk: high | -0.01 | -0.09 | 0.932 | 0.00 |  |  |  |  |
|  | Time: async × ED risk: high | 0.02 | 0.32 | 0.747 | 0.00 |  |  |  |  |
| **Head dissatisfaction** |  |  |  |  |  | 76.81 | 8.89 | 0.92 | 0.21 |
|  | Time: sync | -0.69 | -1.81 | 0.071 | 0.00 |  |  |  |  |
|  | Time: async | -0.42 | -1.11 | 0.269 | 0.00 |  |  |  |  |
|  | ED risk: high | 3.65 | 2.74 | **0.007** | 0.01 |  |  |  |  |
|  | Age | -0.07 | -0.67 | 0.505 | 0.00 |  |  |  |  |
|  | BMI | 0.09 | 0.58 | 0.563 | 0.00 |  |  |  |  |
|  | Ethnicity: Asian | -0.20 | -0.16 | 0.875 | 0.00 |  |  |  |  |
|  | Alexithymia | 0.28 | 5.10 | **<0.001** | 0.10 |  |  |  |  |
|  | Model number | -0.99 | -2.21 | **0.028** | 0.02 |  |  |  |  |
|  | Time: sync × ED risk: high | 2.14 | 3.79 | **<0.001** | 0.00 |  |  |  |  |
|  | Time: async × ED risk: high | 2.02 | 3.58 | **<0.001** | 0.00 |  |  |  |  |
| **Body dissatisfaction** |  |  |  |  |  | 81.08 | 5.92 | 0.95 | 0.24 |
|  | Time: sync | -0.81 | -2.61 | **0.009** | 0.00 |  |  |  |  |
|  | Time: async | -0.68 | -2.19 | **0.029** | 0.00 |  |  |  |  |
|  | ED risk: high | 6.08 | 4.52 | **<0.001** | 0.03 |  |  |  |  |
|  | Age | -0.11 | -1.03 | 0.305 | 0.00 |  |  |  |  |
|  | BMI | 0.41 | 2.47 | **0.014** | 0.03 |  |  |  |  |
|  | Ethnicity: Asian | -1.80 | -1.36 | 0.177 | 0.01 |  |  |  |  |
|  | Alexithymia | 0.22 | 3.96 | **<0.001** | 0.06 |  |  |  |  |
|  | Model number | -0.33 | -0.73 | 0.468 | 0.00 |  |  |  |  |
|  | Time: sync × ED risk: high | 1.69 | 3.66 | **<0.001** | 0.00 |  |  |  |  |
|  | Time: async × ED risk: high | 1.38 | 2.99 | **0.003** | 0.00 |  |  |  |  |
| **Dysmorphic concern** |  |  |  |  |  | 77.20 | 11.05 | 0.91 | 0.29 |
|  | Time: sync | -1.54 | -3.65 | **<0.001** | 0.00 |  |  |  |  |
|  | Time: async | -0.91 | -2.16 | **0.031** | 0.00 |  |  |  |  |
|  | ED risk: high | 8.97 | 6.63 | **<0.001** | 0.07 |  |  |  |  |
|  | Age | -0.20 | -1.94 | 0.054 | 0.02 |  |  |  |  |
|  | BMI | 0.11 | 0.65 | 0.517 | 0.00 |  |  |  |  |
|  | Ethnicity: Asian | -4.30 | -3.28 | **0.001** | 0.04 |  |  |  |  |
|  | Alexithymia | 0.15 | 2.80 | **0.006** | 0.03 |  |  |  |  |
|  | Model number | -0.33 | -0.74 | 0.460 | 0.00 |  |  |  |  |
|  | Time: sync × ED risk: high | 1.53 | 2.43 | **0.016** | 0.00 |  |  |  |  |
|  | Time: async × ED risk: high | 0.63 | 1.01 | 0.315 | 0.00 |  |  |  |  |

Note. Mixed-effects models including model number as a covariate. Model number coded as 1–6 for White participants and 7–10 for East/Southeast Asian participants. ED = Eating disorder; *b* = regression coefficient. Reference categories (time = asynchronous [for H1 models], baseline [for H2-H3 models]; ED risk = low; ethnicity = Caucasian) were assigned a value of 0; whilst categories shown in the table (time = synchronous; ED risk = high; ethnicity = Asian) were assigned a value of 1. b = unstandardized coefficient; SE = standard error; t = t-statistic; p = p-value; semi-partial R² = unique variance explained by model number. Significant *p* values bolded.

**Table S4**

*Multilevel Modelling Results for Main Outcome Variables with Additional Covariates: Model’s Desirability (H1) and Attractiveness (H2-H3)*

| Outcome variable | Fixed effects | *b* | *t* | *p* | Random effects (variance) | Conditional *R*² | Marginal *R*² |
| --- | --- | --- | --- | --- | --- | --- | --- |
| Subjective enfacement (total score) |  |  |  |  | Intercept: 1.02, residual: 0.51 | 0.72 | 0.15 |
|  | Time: sync | 0.60 | 2.53 | **0.012** |  |  |  |
|  | ED risk: high | 0.11 | 0.53 | 0.595 |  |  |  |
|  | Age | -0.03 | -1.88 | 0.062 |  |  |  |
|  | BMI | -0.04 | -1.82 | 0.070 |  |  |  |
|  | Ethnicity: Asian | -0.69 | -3.80 | **<0.001** |  |  |  |
|  | Alexithymia | 0.00 | 0.35 | 0.727 |  |  |  |
|  | Perceived model desirability | 0.14 | 2.62 | **0.010** |  |  |  |
|  | Time: sync × ED risk: high | -0.19 | -1.25 | 0.213 |  |  |  |
| **Objective enfacement** |  |  |  |  | Intercept: 173.15, residual: 55.79 | 0.77 | 0.04 |
|  | Time: baseline | 5.68 | 2.29 | 0.023 |  |  |  |
|  | Time: sync | -3.30 | -1.33 | 0.186 |  |  |  |
|  | ED risk: high | -0.15 | -0.06 | 0.953 |  |  |  |
|  | Age | -0.13 | -0.64 | 0.525 |  |  |  |
|  | BMI | 0.28 | 0.97 | 0.332 |  |  |  |
|  | Ethnicity: Asian | 2.25 | 1.01 | 0.314 |  |  |  |
|  | Alexithymia | 0.03 | 0.33 | 0.743 |  |  |  |
|  | Perceived model desirability | -0.24 | -0.35 | 0.725 |  |  |  |
|  | Time: baseline × ED risk: high | -0.57 | -0.35 | 0.724 |  |  |  |
|  | Time: sync × ED risk: high | 2.07 | 1.28 | 0.203 |  |  |  |
| **Facial attractiveness** |  |  |  |  | Intercept: 1.64, residual: 0.27 | 0.88 | 0.11 |
|  | Time: sync | 0.14 | 0.93 | 0.354 |  |  |  |
|  | Time: async | -0.06 | -0.38 | 0.704 |  |  |  |
|  | ED risk: high | -0.53 | -2.72 | **0.007** |  |  |  |
|  | Age | -0.01 | -0.53 | 0.594 |  |  |  |
|  | BMI | 0.00 | 0.12 | 0.907 |  |  |  |
|  | Ethnicity: Asian | 0.36 | 1.84 | 0.067 |  |  |  |
|  | Alexithymia | -0.02 | -2.71 | **0.007** |  |  |  |
|  | Perceived model attractiveness | -0.00 | -0.07 | 0.945 |  |  |  |
|  | Time: sync × ED risk: high | -0.07 | -0.76 | 0.446 |  |  |  |
|  | Time: async × ED risk: high | -0.02 | -0.16 | 0.872 |  |  |  |
| **Facial adiposity** |  |  |  |  | Intercept: 0.64, residual: 0.17 | 0.82 | 0.12 |
|  | Time: sync | -0.02 | -0.15 | 0.879 |  |  |  |
|  | Time: async | -0.04 | -0.35 | 0.728 |  |  |  |
|  | ED risk: high | 0.28 | 2.19 | **0.029** |  |  |  |
|  | Age | 0.00 | 0.51 | 0.608 |  |  |  |
|  | BMI | 0.04 | 2.57 | **0.011** |  |  |  |
|  | Ethnicity: Asian | -0.13 | -1.02 | 0.310 |  |  |  |
|  | Alexithymia | 0.01 | 2.94 | **0.004** |  |  |  |
|  | Perceived model attractiveness | 0.00 | 0.09 | 0.933 |  |  |  |
|  | Time: sync × ED risk: high | -0.01 | -0.08 | 0.936 |  |  |  |
|  | Time: async × ED risk: high | 0.02 | 0.32 | 0.746 |  |  |  |
| **Head dissatisfaction** |  |  |  |  | Intercept: 77.42, residual: 8.85 | 0.92 | 0.21 |
|  | Time: sync | -2.84 | -3.29 | **0.001** |  |  |  |
|  | Time: async | -2.45 | -2.84 | **0.005** |  |  |  |
|  | ED risk: high | 3.86 | 2.91 | **0.004** |  |  |  |
|  | Age | -0.06 | -0.57 | 0.567 |  |  |  |
|  | BMI | 0.05 | 0.28 | 0.777 |  |  |  |
|  | Ethnicity: Asian | -0.07 | -0.05 | 0.959 |  |  |  |
|  | Alexithymia | 0.26 | 4.82 | **<0.001** |  |  |  |
|  | Perceived model attractiveness | -0.54 | -1.59 | 0.114 |  |  |  |
|  | Time: sync × ED risk: high | 2.16 | 3.83 | **<0.001** |  |  |  |
|  | Time: async × ED risk: high | 2.03 | 3.60 | **<0.001** |  |  |  |
| **Body dissatisfaction** |  |  |  |  | Intercept: 79.27, residual: 6.02 | 0.95 | 0.25 |
|  | Time: sync | -2.59 | -3.65 | **<0.001** |  |  |  |
|  | Time: async | -2.09 | -2.94 | **0.003** |  |  |  |
|  | ED risk: high | 5.97 | 4.52 | **<0.001** |  |  |  |
|  | Age | -0.09 | -0.91 | 0.366 |  |  |  |
|  | BMI | 0.37 | 2.21 | **0.028** |  |  |  |
|  | Ethnicity: Asian | -2.47 | -1.85 | 0.066 |  |  |  |
|  | Alexithymia | 0.22 | 3.98 | **<0.001** |  |  |  |
|  | Perceived model attractiveness | -0.75 | -2.18 | **0.031** |  |  |  |
|  | Time: sync × ED risk: high | 1.79 | 3.85 | **<0.001** |  |  |  |
|  | Time: async × ED risk: high | 1.41 | 3.05 | **0.002** |  |  |  |
| **Dysmorphic concern** |  |  |  |  | Intercept: 74.92, residual: 11.01 | 0.91 | 0.31 |
|  | Time: sync | -3.08 | -3.20 | **0.001** |  |  |  |
|  | Time: async | -1.58 | -1.64 | 0.102 |  |  |  |
|  | ED risk: high | 9.27 | 7.01 | **<0.001** |  |  |  |
|  | Age | -0.22 | -2.11 | **0.036** |  |  |  |
|  | BMI | 0.14 | 0.89 | 0.374 |  |  |  |
|  | Ethnicity: Asian | -2.94 | -2.23 | **0.026** |  |  |  |
|  | Alexithymia | 0.14 | 2.69 | **0.008** |  |  |  |
|  | Perceived model attractiveness | 0.83 | 2.46 | **0.015** |  |  |  |
|  | Time: sync × ED risk: high | 1.54 | 2.46 | **0.014** |  |  |  |
|  | Time: async × ED risk: high | 0.67 | 1.06 | 0.289 |  |  |  |

*Note.* ED = Eating disorder; *b* = regression coefficient. Reference categories (time = asynchronous [for H1 models], baseline [for H2-H3 models]; ED risk = low; ethnicity = Caucasian) were assigned a value of 0; whilst categories shown in the table (time = synchronous; ED risk = high; ethnicity = Asian) were assigned a value of 1. Perceived model desirability: Total sample (*M* = 1.95, *SD* = 1.66); high ED risk (*M* = 2.26, *SD* = 2.01); low ED risk (*M* = 1.52, *SD* = 1.15). Perceived model facial attractiveness: Total sample (*M* = -0.39, *SD* = 1.87); high ED risk (*M* = -0.46, *SD* = 1.98); low ED risk (*M* = -0.34, *SD* = 1.78). Significant *p* values bolded.

**Table S5**

### *Post-Hoc Comparisons by Eating Disorder Risk Group with Additional Covariates: Model’s Desirability (H1) and Attractiveness (H2-H3)*

| **Outcome variable** | **ED risk** | **Comparison** | **MD** | t | *p* |
| --- | --- | --- | --- | --- | --- |
| **Subjective enfacement** | **Low** | Time: async - sync | -0.34 | -3.61 | **0.000** |
|  | **High** | Time: async - sync | -0.37 | -3.50 | **0.001** |
| **Body dissatisfaction** | **Low** | Time: baseline - sync | 0.81 | 2.589 | 0.102 |
|  |  | Time: baseline - async | 0.68 | 2.175 | 0.252 |
|  | **High** | Time: baseline - sync | -0.98 | -2.855 | **0.051** |
|  |  | Time: baseline - async | -0.74 | -2.141 | 0.268 |
| **Dysmorphic concern** | **Low** | Time: baseline - sync | 1.54 | 3.655 | **0.004** |
|  |  | Time: baseline - async | 0.91 | 2.162 | 0.258 |
|  | **High** | Time: baseline - sync | 0.00 | 0.000 | 1.000 |
|  |  | Time: baseline - async | 0.25 | 0.527 | 0.995 |

*Note*. Post hoc analyses were run for models with significant covariates. Models controlled for average levels of perceived model desirably (*M* = 1.95) (H1) or perceived model facial attractiveness (*M* = -0.39) (H2-H3).
